# *Borrelia burgdorferi* Sensu Lato Spirochetes in Wild Birds in Northwestern California: Associations with Ecological Factors, Bird Behavior and Tick Infestation

**DOI:** 10.1371/journal.pone.0118146

**Published:** 2015-02-25

**Authors:** Erica A. Newman, Lars Eisen, Rebecca J. Eisen, Natalia Fedorova, Jeomhee M. Hasty, Charles Vaughn, Robert S. Lane

**Affiliations:** 1 Energy and Resources Group, University of California, 310 Barrows Hall, Berkeley, CA 94720, United States of America; 2 Department of Environmental Science, Policy, and Management, University of California, 130 Mulford Hall, Berkeley, CA 94720, United States of America; 3 Department of Microbiology, Immunology, and Pathology, Colorado State University, Fort Collins, Colorado 80523, United States of America; 4 Division of Vector-Borne Diseases, Centers for Disease Control and Prevention, Fort Collins, Colorado 80522, United States of America; 5 Hawaii Department of Health, Sanitation Branch, Vector Control, Honolulu, Hawaii 96813, United States of America; 6 University of California Hopland Research & Extension Center, Hopland, CA 95449, United States of America; University of Kentucky College of Medicine, UNITED STATES

## Abstract

Although *Borrelia burgdorferi* sensu lato (s.l.) are found in a great diversity of vertebrates, most studies in North America have focused on the role of mammals as spirochete reservoir hosts. We investigated the roles of birds as hosts for subadult *Ixodes pacificus* ticks and potential reservoirs of the Lyme disease spirochete *B. burgdorferi* sensu stricto (s.s.) in northwestern California. Overall, 623 birds representing 53 species yielded 284 *I. pacificus* larvae and nymphs. We used generalized linear models and zero-inflated negative binomial models to determine associations of bird behaviors, taxonomic relationships and infestation by *I. pacificus* with borrelial infection in the birds. Infection status in birds was best explained by taxonomic order, number of infesting nymphs, sampling year, and log-transformed average body weight. Presence and counts of larvae and nymphs could be predicted by ground- or bark-foraging behavior and contact with dense oak woodland. Molecular analysis yielded the first reported detection of *Borrelia bissettii* in birds. Moreover, our data suggest that the Golden-crowned Sparrow (*Zonotrichia atricapilla*), a non-resident species, could be an important reservoir for *B. burgdorferi* s.s. Of 12 individual birds (9 species) that carried *B. burgdorferi* s.l.-infected larvae, no birds carried the same genospecies of *B. burgdorferi* s.l. in their blood as were present in the infected larvae removed from them. Possible reasons for this discrepancy are discussed. Our study is the first to explicitly incorporate both taxonomic relationships and behaviors as predictor variables to identify putative avian reservoirs of *B. burgdorferi* s.l. Our findings underscore the importance of bird behavior to explain local tick infestation and *Borrelia* infection in these animals, and suggest the potential for bird-mediated geographic spread of vector ticks and spirochetes in the far-western United States.

## Introduction

Lyme disease is the most prevalent vector-borne disease in the United States, with 20,000 to 30,000 confirmed cases reported annually from 2003–2011 [1]. Worldwide, clinical manifestations are caused by a subset of genospecies within the expanding *Borrelia burgdorferi* sensu lato (s.l.) spirochete complex [2,3]. These bacteria are transmitted by ticks in the genus *Ixodes*. Larval or nymphal (subadult) ticks ingest spirochetes while feeding upon a bacteremic host, and may then infect other vertebrate hosts while feeding during the subsequent nymphal or adult stages. In North America, Lyme disease is caused by *B*. *burgdorferi* sensu stricto (s.s.). The primary bridging vectors to humans are the nymphal stages of the black-legged tick, *Ixodes scapularis*, in the east, and the western black-legged tick, *Ixodes pacificus*, in the far west [4–6].

In California, *I*. *pacificus* is known to infest >100 species of lizards, birds or mammals [7]. The dusky-footed woodrat (*Neotoma fuscipes*) and the western gray squirrel (*Sciurus griseus*) serve as primary reservoirs for *B*. *burgdorferi* s.l. [8–12], but little is known about the importance of non-mammalian tick hosts, particularly birds, as spirochete reservoirs. Studies attempting to identify potential avian reservoirs of *B*. *burgdorferi* s.l., based on tick loads or infection in birds or their attached ticks, have been conducted in the far-western United States [13–21], as well as in the eastern United States, Europe and Asia (e.g., [22–26]). Birds are potentially important spirochete reservoirs because they are species-rich and ecologically diverse, abundant, occur in the same habitats utilized by the primary mammalian reservoirs, are often infested by vector ticks, and are widely found to carry *B*. *burgdorferi* s.l. (e.g., [27–30]). Birds therefore may be important local reservoirs for various *B*. *burgdorferi* s.l. spirochetes [29,31–34]. However, the ability of spirochete-infected birds to infect feeding ticks is not well understood and varies among bird species [29,35].

Moreover, birds may play an important role in the geographic spread of ticks and borreliae. Dispersal distances for birds are generally much larger than for small mammalian hosts of *Ixodes* ticks, and because of their annual migrations, birds have been implicated as long-distance dispersal agents of vector ticks and pathogenic borreliae (e.g. [22,36]). Bird communities also are changing radically due to human impacts, with large shifts in species composition, total abundance, and relative abundances among species. Geographic distributions of individual species are changing with climate [37,38], land use [39] and introduction of exotic species [40]. Although Lyme disease cases in the far western United States are rare compared to other regions of North America, birds, because of their high mobility, are likely to be the most important vertebrate hosts of *B*. *burgdorferi* s.l. in determining the northward spread of Lyme disease in western North America due to climate and related anthropogenic changes.

Although the primary reservoirs of *B*. *burgdorferi* s.l. in the United States are mammals, there is growing evidence that different vertebrate species contribute to the enzootic maintenance of Lyme disease spirochetes in the far west. As birds are species-rich (433 regularly-occurring species in California [41]) and their community composition varies greatly among California’s diverse vegetation communities, clarifying the role of birds as reservoirs for *B*. *burgdorferi* s.l. is important both for more accurate modeling of the local ecology of Lyme disease spirochetes and to better understand the risk of human exposure to infected ticks.

The goals of this study are to identify bird species that are potentially important to the enzootic maintenance of Lyme disease spirochetes in northwestern California, to evaluate the individual nesting and feeding behaviors of birds that contribute to the transmission of spirochetes, and to test for the first time whether avian taxonomic relationships are predictive of *I*. *pacificus* larval and nymphal loads, and of *B*. *burgdorferi* s.l. infection.

## Methods

### Site description

The study was conducted at 14 sites within the 2,168-ha (5,358-ac) University of California, Hopland Research and Extension Center (HREC) (39°00’03 N, 123°04’59 W), located in the foothills of the Mayacmas Mountains (often alternately spelled as “Mayacamas”) in Mendocino County in northwestern California. The HREC ranges in elevation from 152–914 m (500–3000 ft). The region’s climate can be characterized as Mediterranean, with hot, dry summers and cool, wet winters. Average yearly temperature is 14°C (57°F), with maximum monthly mean temperatures of 13°C (56°F) in the winter and 33°C (91°F) in the summer. The average annual rainfall is 940 mm (37 in) (HREC, published online at http://hrec.ucanr.edu/Weather,_Physical,_and_Biological_Data/). The four dominant plant communities are dense oak woodland including Pacific madrone (*Arbutus menziesii*) with an understory mainly composed of leaf litter; oak-woodland communities with sparse oaks surrounded by grass and herbaceous forbs; open grasslands; and a diverse chamise-type chaparral (*Adenostoma fasciculatum*) community containing significant amounts of California lilac (*Ceanothus* spp.) and manzanita (*Arctostaphylos* spp.). Oak species at HREC include interior live oak (*Quercus wislizeni*), coast live oak (*Quercus agrifolia*), valley oak (*Quercus lobata*), California black oak (*Quercus kelloggii*), and blue oak (*Quercus douglasii*). Over two hundred species of birds and 50 species of mammals have been reported from HREC (http://ucanr.edu/sites/hopland/Natural_Resources/). Primary vertebrate reservoirs of *B*. *burgdorferi* s.l. at HREC include the western gray squirrel and the dusky-footed woodrat, and zooprophylactic (refractory) hosts include the southern alligator lizard (*Elgaria multicarinata*) and the western fence lizard (*Sceloporus occidentalis*) [8–12,42,43].

### Bird sampling

Bird capture methods were described by Eisen et al. [16]. In brief, birds were captured by mist netting during the breeding seasons of 2003 and 2004. Mist netting occurred over four-day sampling periods at 14 sites during >20 sessions per year from March through July (nets measuring 9–12 m, 30 mm mesh size). Although nymphs of *I*. *pacificus* have been found in low numbers in all seasons, the main period of nymphal activity in Mendocino County lasts from March through June or July, with peak numbers from late April to late May [44,45]. In 2003, five nets were placed at each site to maximize capture success, with two nets sited in dense oak woodland, one net placed either in chaparral or grassland, and two nets sited along the ecotone between habitats (sampling the oak woodland community). In 2004, mist netting occurred at 3 of the 13 original sites, and one new site. Potter ground traps baited with birdseed were also used (4-cell Potter traps; Boreal King Supply Co., Bigfork, Minnesota). Nets and ground traps were opened within 15 min of sunrise and kept open for 4–8 h, depending on daily environmental conditions and capture success.

### Bird and tick processing

Birds were identified to species and examined for presence of ticks, particularly around the eyes and beak where most ixodid ticks attach. All ticks found were removed with fine-tipped forceps. Search time was not standardized because of variation in the birds’ sizes, behavior of different individuals when handled (calm or not), and because of the time involved in removing attached ticks. Blood was drawn from the bird’s ulnar vein, and stored in EDTA-coated blood storage tubes at-80 C°. A tail feather was clipped to indicate when a bird was recaptured within a season. All handling and sampling of birds were conducted following protocols approved by the Institutional Animal Care and Use Committee at the University of California at Berkeley and the California Department of Fish and Game.

Ticks were preserved in 95% ethanol. A stereomicroscope at 30–40x magnification was used to distinguish *I*. *pacificus* larvae from those of *Ixodes woodi* and *Ixodes spinipalpis*. Larvae not easily identified as either *I*. *pacificus* or *I*. *spinipalpis* were treated for 1 h in 10% potassium hydroxide, mounted in Hoyer’s medium on a microscope slide, and examined by light microscopy at magnifications up to 400x.

### Taxonomy and nomenclature of Borrelia

The *B*. *burgdorferi* s.l. (BBSL) complex consists of 20 confirmed or proposed genospecies [2,3], at least six of which (*B*. *americana*, *B*. *bissettii* (BBIS), *B*. *burgdorferi* s.s. (BBSS), *B*. *californiensis*, *B*. *carolinensis*, and *B*. genomospecies 2 [sensu Postic et al. [46]]) occur in California. BBSS is of special interest because it causes Lyme disease, and BBIS has been repeatedly detected in or isolated from California mammals [11], and occasionally, humans [47]. We use the code BBSL(un) to refer to undifferentiated genospecies in BBSL that are neither BBSS nor BBIS, and use the term BBSLmix to refer to mixed infections detected in a single sample.

### Tissue DNA extraction, PCR and sequencing

Genomic DNA from ticks and for bird blood samples was extracted using DNeasy Blood and Tissue Kit (Qiagen, Valencia, CA) according to the manufacturer’s protocol as described previously [48,49]. All DNA extracts were tested with a nested polymerase chain reaction (PCR) targeting the 5S-23S rRNA intergenic spacer (IGS) of BBSL. Primers and the PCR protocol used were described previously by Lane et al. [48]. Due to its nucleated erythrocytes, bird blood contains large amounts of DNA and presents challenges for the detection of *Borrelia* DNA, which has far fewer copies per sample than does bird DNA. Blood DNA extracts were first screened using the Lane et al. [48] PCR protocol, but because of inconsistent results, the protocol was optimized and all samples were retested. The PCR protocol was modified by (a) adjusting the concentration of the Amplitaq DNA polymerase buffer (Life Technologies, Grand Island, New York) from 10x to 12x; (b) decreasing the denaturation temperature to 88°C; and (c) increasing the annealing time to 2 min and the annealing temperature to 55°C in the first round of amplification. Each PCR run contained multiple wells of *B*. *burgdorferi* strain CA4 and UV-treated nuclease free water as positive and negative controls, respectively. PCR products were electrophoresed with 1.5% agarose gels, stained with ethidium bromide, and visualized by UV transillumination. All bird blood DNA extracts were tested at least twice in separate runs, and if results were in disagreement, a third test was performed. Samples that tested positive twice were considered to be positive. Products from positive samples were purified using QIAquick PCR Purification Kit (Qiagen, Valencia, CA). Sequencing was performed at the University of California, Berkeley DNA Sequencing Facility using inner PCR primers [48]. Sequences were assembled and manually edited using Sequencher 4.6 (Gene Codes Corp., Ann Arbor, MI). Contigs with more than one nucleotide at the same position in forward and reverse directions were considered mixed infections (BBSLmix). Each sequence was directly compared with sequences of the same loci from various spirochete genospecies available in the GenBank database using the Basic Local Alignment Search Tool (http://www.ncbi.nlm.nih.gov/BLAST/). The criterion for inclusion within a *Borrelia* species was ≥99% similarity. Data for the 5S-23S rRNA IGS DNA sequences have been deposited in Dryad, in FASTA format.

### Modeling infection and infestation: modeling goals

Explanatory variables, including ecological and taxonomic variables, were evaluated in models of BBSL infection status in birds or attached *I*. *pacificus* larvae, and in modeling presence of larvae and of nymphs on birds using bionomial models in a generalized linear modeling (GLM) framework. The same variables were used to predict *I*. *pacificus* larval and nymphal loads on birds using zero-inflated models for count data. Life-history traits included as explanatory variables were log-transformed average body weight for each bird species [50–52], resident status, breeding status, feeding substrate, main food item, main habitat type, and nest placement [51]. For modeling infection, we also used the number of nymphs on the individual bird and year as explanatory variables. We attempted to control for differences in immune system with the taxonomic variables order, family, and genus [53]. “Species” was also investigated as an explanatory variable with 52 levels, but could not be included in any model given the generally low sample sizes.

Bird taxonomic relationships are presented in Table 1. Bird species’ preferences for a main habitat were derived from the Cornell Lab of Ornithology online, and then matched to the four main habitat categories reported from the HREC: chaparral, grass, oak woodland-grass, and dense oak woodland. Body weights for birds by species were also taken from the Cornell Lab of Ornithology online, and where ranges of body weights were provided, an arithmetic mean was calculated and used in each analysis (see Table A in S1 File). The resident status of birds was determined by long-term HREC observations [54]. Explanatory variables and their levels are summarized in Table 2, and bird species categorized by ecological guilds are listed in Table A in S1 File. Ecological variables included foraging and other behaviors based on classifications presented in De Graaf [55], and modified by the Cornell Lab of Ornithology [51].

**Table 1:** Bird taxonomic hierarchy and 4-letter codes for species identification.

Order	Family	Scientific name	Common name	4-Letter Code
Galliformes	Phasianidae	*Callipepla californica*	California Quail	CAQU
Passeriformes	Aegithalidae	*Psaltriparus minimus*	Bushtit	BUSH
	Cardinalidae	*Passerina amoena*	Lazuli Bunting	LAZB
		*Pheucticus melanocephalus*	Black-headed Grosbeak	BHGR
	Certhiidae	*Certhia americana*	Brown Creeper	BRCR
	Corvidae	*Aphelocoma californica*	Western Scrub-Jay	WESJ
		*Cyanocitta stelleri*	Steller’s Jay	STJA
	Emberizidae	*Aimophila ruficeps*	Rufous-crowned Sparrow †	RCSP
		*Artemisiospiza belli (Amphispiza belli)*	Bell’s Sparrow **	SAGS
		*Chondestes grammacus*	Lark Sparrow	LASP
		*Junco hyemalis*	Dark-eyed Junco	DEJU
		*Melospiza lincolnii*	Lincoln's Sparrow	LISP
		*Melospiza melodia*	Song Sparrow	SOSP
		*Passerella iliaca*	Fox Sparrow	FOSP
		*Pipilo crissalis*	California Towhee *	CALT
		*Pipilo maculatus*	Spotted Towhee	SPTO
		*Spizella passerina*	Chipping Sparrow	CHSP
		*Zonotrichia atricapilla*	Golden-crowned Sparrow	GCSP
	Fringillidae	*Haemorhous purpureus (Carpodacus purpureus)*	Purple Finch	PUFI
	Hirundinidae	*Tachycineta thalassina*	Violet-green Swallow	VGSW
	Icteridae	*Agelaius phoeniceus*	Red-winged blackbird	RWBB
		*Icterus bullockii*	Bullock's Oriole	BUOR
		*Molothrus ater*	Brown-headed Cowbird	BHCO
	Mimidae	*Toxostoma redivivum*	California Thrasher *	CATH
	Paridae	*Baeolophus inornatus*	Oak Titmouse *	OATI
	Parulidae	*Dendroica coronata*	Yellow-rumped Warbler	YRWA
		*Dendroica nigrescens*	Black-throated Gray Warbler	BTYW
		*Vermivora celata*	Orange-crowned Warbler	OCWA
		*Vermivora ruficapilla*	Nashville Warbler	NAWA
	Passeridae	*Spinus psaltria (Carduelis psaltria)*	Lesser Goldfinch	LEGO
	Sittidae	*Sitta carolinensis*	White-breasted Nuthatch	WBNU
	Sturnidae	*Sturnus vulgaris*	European Starling	EUST
	Sylviidae	*Polioptila caerulea*	Blue-gray Gnatcatcher	BGGN
	Thraupidae	*Piranga ludoviciana*	Western Tanager	WETA
	Timaliidae	*Chamaea fasciata*	Wrentit *	WREN
	Troglodytidae	*Thryomanes bewickii*	Bewick's Wren	BEWR
		*Troglodytes aedon*	House Wren	HOWR
	Turdidae	*Catharus ustulatus*	Swainson's Thrush	SWTH
		*Sialia mexicana*	Western Bluebird	WEBL
		*Turdus migratorius*	American Robin	AMRO
	Tyrannidae	*Contopus sordidulus*	Western Wood-Pewee	WEWP
		*Empidonax difficilis*	Pacific-slope Flycatcher	PSFL
		*Myiarchus cinerascens*	Ash-throated Flycatcher	ATFL
		*Sayornis nigricans*	Black Phoebe	BLPH
	Vireonidae	*Vireo cassinii*	Cassin's Vireo	CAVI
		*Vireo gilvus*	Warbling Vireo	WAVI
		*Vireo huttoni*	Hutton's Vireo	HUVI
		*[Vireo olivaceus]*	[Red-eyed Vireo] ‡	[REVI]
Piciformes	Picidae	*Colaptes auratus*	Northern Flicker	NOFL
		*Melanerpes formicivorus*	Acorn Woodpecker	ACWO
		*Picoides nuttallii*	Nuttall's Woodpecker *	NUWO
		*Picoides pubescens*	Downy Woodpecker	DOWO
Strigiformes	Strigidae	*Otus kennicottii*	Western Screech-Owl	WESO

* Indicates a species endemic or near-endemic to the California Floristic Province (CFP)

** Species that breeds in this region is endemic to the CFP was recently promoted from the subspecies Bell’s Sage Sparrow (*Artemisiospiza belli belli*) to full species status (*Artemisiospiza belli*) [53]

† Natural history data not available from Cornell Lab of Ornithology Online. Data instead taken from Birds of North America online [52]

‡ Accidental bird not normally found in region

(Names in round parentheses) are previous taxonomic designations

[Species in square parentheses] excluded from training data

**Table 2:** Explanatory variables and their levels included in binomial and zero-inflated models.

Explanatory variable	Levels (or data type)	Level names
log(average body weight) (LOG.AVEBWT)	(continuous)	(continuous)
Breeding status (BRD)	breeding	BREED
	non-breeding	NONBRD
Feeding substrate/behavior (FDSUB)	air (aerial, flycatching, hovering)	AIR
	bark	BARK
	foliage gleaning	FOLIAGE
	ground	GROUND
	stalking	STALK
Main food (MNFD)	insects	INSECT
	omnivore	OMNIVORE
	mammals	MAMMAL
	seeds	SEEDS
Main habitat (MNHAB)	chaparral	CHAP
	grass	GRASS
	oak woodland-grass	OAKW
	dense oak woodland	XW
Nest substrate (NEST)	bark	BARK
	cavity	CAVITY
	ground	GROUND
	opportunistic	OPP
	shrub	SHRUB
	tree	TREE
	non-breeding	NON
Resident status (RESSTAT)	resident	RES
	non-resident	NONRES
Year (YEAR)	2 levels by data collection date	2003
		2004
Number of nymphs removed from bird (N_NYM)	(count data)	(count data)
Species (SPECIES)	52 instances by 4-letter species codes	[52 levels (excludes REVI)]
Genus (GENUS)	44 instances by genus name	[44 levels] (see Table 1)
Family (FAMILY)	24 instances by family name	[24 levels] (see Table 1)
Order (ORDER)	4 instances by order name	[4 levels] (see Table 1)

We chose specific modeling frameworks (including selection of link functions) after performing extensive preliminary analyses. After zero-truncating the dataset such that only positive results (non-zero instances of bird infection, or larval or nymphal presence, depending on analysis) were examined, we found that in each case, data were not normally distributed, and also violated assumptions of homoscedasticity of errors. Additionally, bird individuals per species varied, leading to “unbalanced” data structure for each analysis. These issues are inherent to multiple-species studies that examine parasite loads and require appropriate models that can model both zero-inflated data and account for unbalanced data.

For these reasons, a bionomial model in a GLM framework was appropriate for explaining (1) presence of infection in birds and larvae removed from individual birds, (2) presence of larval ticks on individual birds, and (3) presence of nymphs on individual birds. We used zero-inflated models for count data for (4) number of larvae and (5) number of nymphs removed from individual birds. Zero-inflated negative binomial models used here have two parts: a negative binomial count model with a log link, and a zero-inflation binomial model with a logit link that models the presence of extra zeros. Here, true zeroes represent ecologically meaningful information in that, for example, the bird was inspected, but no larvae or nymphs were found for infestation models. Likewise for infection models, zeroes might represent larvae removed from birds that are tested for but lack infection either due to lack of exposure to an infected bird, or lack of infection due to low level of engorgement and short feeding time prior to testing. Both modeling approaches, that is, binomial models in a GLM framework and zero-inflated negative binomial models, generated model outcomes that were then subjected to model selection based on Akaike Information Criterion (AIC) values [56]. All modeling goals are summarized in Table 3.

**Table 3:** Bird infection and tick infestation modeling goals and best supported models, based on AIC and AICc values.

Modeling goal	Model family	Data	Unit of analysis	Outcome
*Generalized linear models*				
Bird infection (presence/absence)	binomial	orders: Passeriformes and Piciformes	individual bird	Best supported model: Bird infection ~ N_NYM + YEAR + ORDER
Bird infection (presence/absence)	binomial	order: Passeriformes only	individual bird	Best supported model: Bird infection ~ N_NYM +YEAR + LOG_BWT
Larval infestation (presence/absence)	binomial	orders: Passeriformes and Piciformes	individual bird	Best supported model: Larval presence ~ YEAR + ORDER + FDSUB
Larval infestation (presence/absence)	binomial	order: Passeriformes only	individual bird	Best supported model: Larval presence ~ YEAR + NEST + FDSUB
Nymphal infestation (presence/absence)	binomial	orders: Passeriformes and Piciformes	individual bird	Best supported model: Nymphal presence ~ YEAR + FAMILY
Nymphal infestation (presence/absence)	binomial	order: Passeriformes only	individual bird	Best supported model: Nymphal presence ~ YEAR + FAMILY + MNHAB
*Zero-inflated negative binomial models*				
Larval infestation (count data)	(count model: negative binomial with log link) | (zero-inflation model: binomial model with logit link)	orders: Passeriformes and Piciformes	individual bird	Best supported model: Number of larvae ~ (MNHAB) | (LOG.AVEBWT + YEAR)
Nymphal infestation (count data)	(count model: negative binomial with log link) | (zero-inflation model: binomial model with logit link)	orders: Passeriformes and Piciformes	individual bird	Best supported model: Number of nymphs ~ (MNHAB + FDSUB) | (YEAR + FDSUB)
*Body weight Null Models*				
*BNM*: Bird infection	single-variable linear regression	all observations	species	not significant
*BNM*: Bird infection	single-variable linear regression	positive counts only	species	not significant
*BNM*: Larval infestation	single-variable linear regression	all observations	species	not significant
*BNM*: Larval infestation	single-variable linear regression	positive counts only	species	not significant
*BNM*: Nymphal infestation	single-variable linear regression	all observations	species	not significant
*BNM*: Nymphal infestation	single-variable linear regression	positive counts only	species	not significant

### Modeling infection and infestation: explanatory variables

Criteria used to establish that a particular bird species may serve as a *Borrelia* reservoir host were as follows: (a) a bird can obtain spirochetes only from an infected *I*. *pacificus* nymph; (b) the spirochetes must survive in the tissues or blood of a reservoir bird for some period of time; and (c) the reservoir bird must be able to transmit spirochetes to feeding ticks. Criteria (a) and (c) were established based on the prior knowledge that unfed *I*. *pacificus* larvae are BBSL-free (transovarial transmission occurs rarely, if ever); therefore, detection of BBSL in a larva removed from a bird can be assumed to very likely demonstrate infection in that bird as long as no infected nymphs were feeding on the bird at the same time (potentially resulting in co-feeding infection). For tick infestation, modeling was done separately for nymphs and for larvae.

Bird contact with larvae or nymphs may lead to an increase in overall tick abundance in an ecosystem, and contact with nymphs may increase the prevalence of BBSL in bird populations. Using species-level body weight and behavioral data, we hypothesize that:

(1) The number of nymphs on a bird is predictive of BBSL infection status. Because only ~5–15% of host-seeking nymphs in northwestern California carry BBSL [5,16,57], a higher nymphal load should increase the likelihood that a bird is bitten by a BBSL-infected nymph;
(2) Average body weight of birds by species correlate to tick loads and therefore higher likelihood of exposure to BBSL. Body weight may therefore serve as an adequate null model of infestation and infection prevalence, either because of larger surface area of skin, or correlation with life history traits such as home range size [58], ground nesting and foraging behaviors;
(3) Contact with leaf litter through either ground foraging or ground nesting is predictive of tick load for both larvae and nymphs [16,59,60] for individual birds;
(4) Contact with bark from tree bases through feeding or use as nesting substrate is predictive of tick load [16,22,60] for individual birds;
(5) Breeding birds have higher tick loads than non-breeding birds that migrate and/or are winter residents because of increased foraging activity associated with nest-building and feeding their young, and therefore breeding birds will sample more of their surroundings than non-breeding birds;
(6) The main food item of each species of bird (categories include insects, seeds, mammals and “omnivorous”), while defining much about foraging behaviors, is not predictive of tick load in individual birds, because it may be too coarse a category in explaining bird behavior given generalizations about feeding behavior, and due to dietary switching [61];
(7) Year-round residents do not have higher tick loads than migratory birds, because subadult ticks are attached to the birds for <1 week. In our data, it is not the case that all migratory birds arrived one week or less before they were captured. We test this variable because migratory birds live in tropical regions outside the geographic distribution of *I*. *pacificus* ticks for much of the year and there may be a difference between these and resident birds;
(8) Sampling year, as a proxy for interannual variation in environmental variables such as temperature and annual rainfall (both correlated to density of questing ticks), is predictive of the number of larvae and nymphs found on individual birds;
(9) The main habitat of birds by species is predictive of the presence of larvae and nymphs, and of BBSL infection, for individual birds. In the ecosystems studied, dense oak woodland was found to have BBSS-infected nymphs, although the drag-sampling method used to show this is ineffective in grass, chaparral, and grass-understory oak-woodland, which may also harbor BBSS-infected nymphs [62].
(10) Certain taxonomic groups (within family, order or genus) of birds will have more infected members than other taxonomic groups.

None of the birds evaluated exhibit social grooming, so a variable related to this behavior of interest was not included in models. All birds were sampled in or close to their breeding seasons, so their immunological status was assumed to be similar. Because some species could not be accurately sexed in the field, we did not distinguish males from females in our analyses. One accidental bird species (Red-eyed Vireo) is excluded from most analyses (except body weight null models) because its interactions with California ecosystems are unknown. Two birds that were the sole representatives of their taxonomic orders (the Western Screech-Owl and California Quail) were excluded from model analyses. One Brown-headed Cowbird was excluded from the analysis of nesting substrate because of that species’ opportunistic and brood-parasitic behavior, and four Brown Creepers were reclassified as “bark” nesters rather than placed in the suggested “tree” nesting category because of the species’ unique nesting strategy [63].

### Modeling infection and infestation: model specification

We define “infection” as any genospecies of BBSL detected from a bird and/or in a larva found attached to a bird. Bird infection was modeled as a binomial distribution (with dispersion parameter = 1), with the individual bird as a unit of analysis. The dependent variable was constructed as a column vector of “successes” (infected) and “failures” (not infected):

y=c[infected,notinfected] (1)

A full, or maximal model was specified:

y∼LOG.AVEBWT+FDSUB+MAINHAB+MNFD+BRD+RESSTAT+NEST+YEAR+N_NYM+ORDER+FAMILY+GENUS (2)

(with variables and their abbreviations above defined in Table 2) and was reduced to form a candidate set, which were then tested through extensive model comparison with Akaike Information Criterion (AIC) values and second order AIC (AICc) “corrected” values, which assign heavier penalties for additional parameters. Similar analyses were performed for presence of larvae on individual birds, and presence of nymphs on individual birds. Count models for number of larvae and number of nymphs removed from individual birds reduced the same full model above for both the negative binomial count model and the zero-inflation binomial model parts of the zero-inflated negative binomial model.

All analyses above were completed in R (version 3.0.1) using the packages “stats” [64], “pscl” [65,66], and “AICcmodavg” [67].

## Results

### Capture effort and success

Sampling efforts for birds included 3,117 mist net hours at 14 sites and 910 Potter trap hours (at 8 sites only in 2003) for birds. In total, 623 individual birds comprising 4 orders, 24 families, 44 genera and 53 species were captured, all by mist netting. *Ixodes pacificus* made up > 99% (284 of 286) of ticks removed from birds. The remaining two ticks were the rabbit tick, *Haemaphysalis leporispalustrus*.

### Summary statistics of infestation and infection

The majority of birds were not infested with ticks and the majority of examined larval ticks were not infected with BBSL. Data for tick infestation, and tick-infection prevalence were highly zero-inflated. Only 100 (16.1%) of 623 examined birds carried *I*. *pacificus* ticks, with 62 (10.0%) birds infested with a total of 192 larvae and 55 birds (8.8%) infested with 92 nymphs, for a total of 284 ticks. BBSL was detected only from the bird itself for 57 (9.1%) individuals, while 66 birds had BBSL present either in blood and attached larvae or only in attached larvae. Among the four orders of birds represented, BBSL was detected from 23 species, and infected larvae were recovered from 9 species, all in the order Passeriformes. The 23 bird species containing individuals with *Borrelia* present, and the remaining uninfected species, are organized by larval and nymphal tick loads (Table 4). Of 192 larvae tested, 25 (13.0%) were infected. Of 85 nymphs tested, 21 (24.7%) were infected. Seven ticks were not tested because they were either damaged or lost.

**Table 4:** Summary of *Ixodes pacificus* infestation on birds, *Borrelia burgdorferi* s.l. (BBSL) infection in bird blood, and number of BBSL infected larvae by bird species.

4 Letter species code	N birds	N larvae (no. larvae/bird)	N nymphs (no. nymphs/bird)	N birds with BBSL (proportion)	BBSL infected larvae (proportion)
*Birds infested by infected larvae (with or without infection of the birds)*					
CATH	5	1 (0.20)	1 (0.20)	0 (0.00)	1 (1.00)
BUOR	12	1 (0.08)	3 (0.25)	0 (0.00)	1 (1.00)
SPTO	7	1 (0.14)	1 (0.14)	0 (0.00)	1 (1.00)
CHSP	5	3 (0.60)	0 (0.00)	1 (0.20)	1 (0.33)
AMRO	5	7 (1.40)	1 (0.20)	2 (0.40)	2 (0.29)
BEWR	24	5 (0.21)	7 (029)	3 (0.13)	1 (0.20)
LASP	4	96 (24.00)	14 (3.50)	0 (0.00)	14 (0.15)
OCWA	28	7 (0.25)	0 (0.00)	3 (0.11)	1 (0.14)
DEJU	58	22 (0.38)	14 (0.24)	4 (0.07)	3 (0.14)
**Subtotal**	**148**	**143**	**41**	**13**	**25**
*Infected birds without infected larvae*					
RWBB	3	0 (0.00)	0 (0.00)	1 (0.33)	–
WEWP	3	0 (0.00)	0 (0.00)	1 (0.33)	–
GCSP	14	2 (0.14)	0 (0.00)	4 (0.29)	–
STJA	4	7 (1.75)	7 (1.75)	1 (0.25)	–
BRCR	4	3 (0.75)	8 (2.00)	1 (0.25)	–
BHGR	8	0 (0.00)	2 (0.25)	2 (0.25)	–
WETA	4	0 (0.00)	0 (0.00)	1 (0.25)	–
BTYW	9	1 (0.11)	0 (0.00)	2 (0.22)	–
CAVI	10	0 (0.00)	1 (0.10)	2 (0.20)	–
WEBL	14	0 (0.00)	3 (0.21)	2 (0.14)	–
PUFI	13	0 (0.00)	1 (0.08)	2 (0.15)	–
WBNU	8	3 (0.38)	0 (0.00)	1 (0.13)	–
OATI	81	20 (0.25)	14 (0.17)	11 (0.14)	–
CALT	10	1 (0.10)	0 (0.00)	1 (0.10)	–
BUSH	21	0 (0.00)	0 (0.00)	2 (0.10)	–
LEGO	100	0 (0.00)	0 (0.00)	8 (0.08)	–
WAVI	28	2 (0.07)	1 (0.04)	2 (0.07)	–
PSFL	14	0 (0.00)	1 (0.07)	1 (0.07)	–
**Subtotal**	**348**	**39**	**38**	**30**	–
*Non-infected birds carrying ticks*					
HOWR	2	4 (2.00)	8 (4.00)	–	–
WESO	1	1 (1.00)	1 (1.00)	–	–
FOSP	1	1 (1.00)	0 (0.00)	–	–
LISP	1	0 (0.00)	1 (1.00)	–	–
NOFL	4	1 (0.25)	0 (0.00)	–	–
SAGS	4	1 (0.25)	0 (0.00)	–	–
HUVI	6	0 (0.00)	1 (0.17)	–	–
ACWO	19	0 (0.00)	2 (0.11)	–	–
ATFL	13	1 (0.08)	0 (0.00)	–	–
WREN	29	1 (0.03)	0 (0.00)	–	–
**Subtotal**	**80**	**10**	**13**	–	–
*Non-infected birds without ticks*					
BGGN	2	0 (0.00)	0 (0.00)	–	–
BHCO	1	0 (0.00)	0 (0.00)	–	–
BLPH	6	0 (0.00)	0 (0.00)	–	–
CAQU	1	0 (0.00)	0 (0.00)	–	–
DOWO	1	0 (0.00)	0 (0.00)	–	–
EUST	1	0 (0.00)	0 (0.00)	–	–
LAZB	3	0 (0.00)	0 (0.00)	–	–
NAWA	2	0 (0.00)	0 (0.00)	–	–
NUWO	7	0 (0.00)	0 (0.00)	–	–
RCSP	1	0 (0.00)	0 (0.00)	–	–
REVI	1	0 (0.00)	0 (0.00)	–	–
SOSP	8	0 (0.00)	0 (0.00)	–	–
SWTH	1	0 (0.00)	0 (0.00)	–	–
VGSW	9	0 (0.00)	0 (0.00)	–	–
WESJ	2	0 (0.00)	0 (0.00)	–	–
YRWA	1	0 (0.00)	0 (0.00)	–	–
**Subtotal**	**47**	**0**	**0**	–	–
**Total**	**623**	**192**	**92**	**43**	**25**

### BBSL genospecies detected

**Fig 1:**
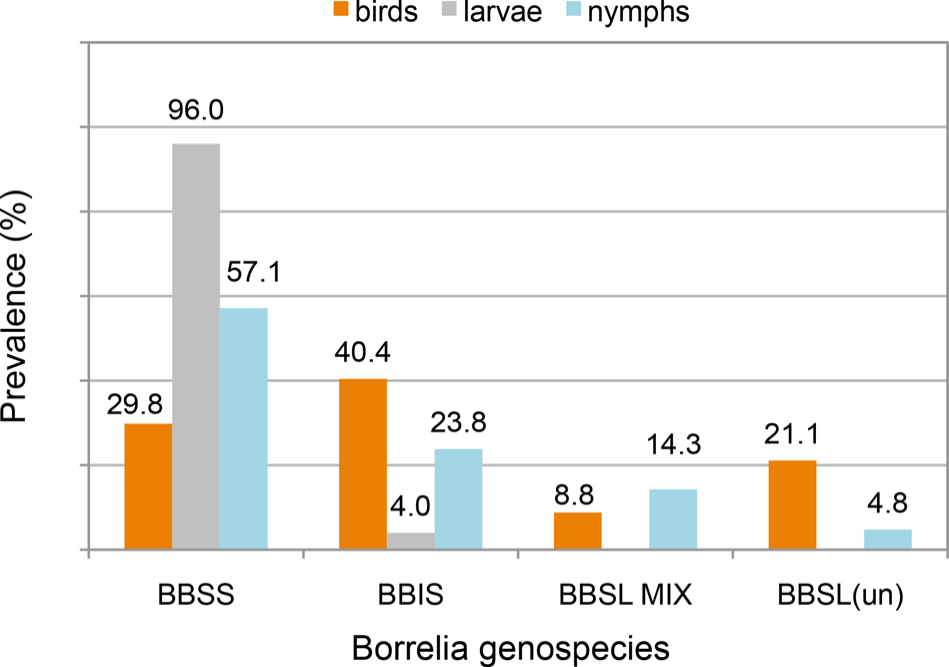
Prevalence (by %) of infection of birds, *I*. *pacificus* larvae and nymphs with different genospecies of *Borrelia*.

The prevalence of BBSL infection differed among samples from birds (57 positive findings/623 specimens; 9.2%), larvae removed from birds (25/192; 13.0%), and nymphs removed from birds (21/92; 22.8%). All four BBSL subcategories—BBSS, BBIS, BBSL(un) and BBSLmix—were detected in birds and nymphs, whereas larvae were infected with only BBSS and BBIS. The representations of subcategories among, respectively, birds, larvae, and nymphs (with the most common genospecies for each in bold) were BBSS (29.8, **96.0, 57.1**), BBIS (**40.4**, 4.0, 23.8), BBSL(un) (21.1, 0.0, 9.5), and BBSLmix (8.8, 0.0, 9.5). See Fig. 1. Data on BBSL diversity in birds, larvae or nymphs are presented in Tables 5–7.

**Table 5:** Summary of *B*. *burgdorferi* s.l. genospecies found in birds.

4 Letter species code	Raw proportion of birds with BBSL (%)	BBSL genospecies			
		BBSS	BBIS	BBSL(un)	BBSLmix
*Birds infested by infected larvae (with or without infection of the birds)*					
CATH	0/5	–	–	–	–
BUOR	0/12	–	–	–	–
SPTO	0/7	–	–	–	–
CHSP	1/5 (20.0)	–	–	1	–
AMRO	2/5 (40.0)	–	2	–	–
BEWR	3/24 (12.5)	1	1	1	–
LASP	0/4	–	–	–	–
OCWA	3/28 (10.7)	1	2	–	–
DEJU	4/58 (6.9)	1	1	1	1
**Subtotal**	**13/148**	**3**	**6**	**3**	**1**
*Infected birds without infected larvae*					
RWBB	1/3 (33.3)	–	1	–	–
WEWP	1/3 (33.3)	–	1	–	–
GCSP	4/14 (28.6)	4	–	–	–
STJA	1/4 (25.0)	–	1	–	–
BRCR	1/4 (25.0)	–	–	1	–
BHGR	2/8 (25.0)	2	–	–	–
WETA	1/4 (25.0)	–	–	1	–
BTYW	2/9 (22.2)	1	–	1	–
CAVI	2/10 (20.0)	1	1	–	–
PUFI	2/13 (15.4)	–	1	1	–
WEBL	2/14 (14.3)	1	–	–	1
WBNU	1/8 (12.5)	–	–	1	–
OATI	11/81 (13.6)	3	4	1	2
CALT	1/10 (10.0)	–	–	1	–
BUSH	2/21 (9.5)	–	1	1	–
LEGO	8/100 (8.0)	2	4	1	1
WAVI	2/28 (7.1)	–	2	–	–
PSFL	1/14 (7.1)	–	1	–	–
**Subtotal**	**44/348**	**14**	**17**	**9**	**4**
*Non-infected birds carrying ticks*					
ACWO	0/19	–	–	–	–
HOWR	0/2	–	–	–	–
HUVI	0/6	–	–	–	–
**Subtotal**	**0/27**	**0**	**0**	**0**	**0**
**Totals**	**57/523**	**17**	**23**	**12**	**5**

Numbers in bold with an asterisk (*) indicate matching genospecies between individual bird and hosted ticks, for the indicated category. Acronyms in table are: BBSL, *B*. *burgdorferi* s.l; BBSS, *B*. *burgdorferi* sensu stricto; BBIS, *B*. *bissettii*; BBSL(un), undifferentiated genospecies in the *B*. *burgdorferi* sensu lato complex that are neither BBSS nor BBIS; and BBSLmix, any combination of BBSL(un), BBSS, and BBIS detected in a single sample.

**Table 6:** Summary of *B*. *burgdorferi* s.l. genospecies found in *I*. *pacificus* larvae removed from birds.

4 Letter species code	Raw proportion of BBSL-infected larvae (%)	BBSL genospecies			
		BBSS	BBIS	BBSL(un)	BBSLmix
*Birds infested by infected larvae (with or without infection of the birds)*					
CATH	1/1 (100.0)	1	–	–	–
BUOR	1/1 (100.0)	1	–	–	–
SPTO	1/1 (100.0)	1	–	–	–
CHSP	1/3 (33.3)	1	–	–	–
AMRO	2/7 (28.6)	2	–	–	–
BEWR	1/5 (20.0)	1	–	–	–
LASP	14/96 (14.6)	14	–	–	–
OCWA	1/7 (14.3)	1	–	–	–
DEJU	3/22 (13.6)	2	1	–	–
**Subtotal**	**25/143**	**24**	**1**	**0**	**0**
*Infected birds without infected larvae*					
RWBB	0	–	–	–	–
WEWP	0	–	–	–	–
GCSP	0/2	–	–	–	–
STJA	0/7	–	–	–	–
BRCR	0/3	–	–	–	–
BHGR	0	–	–	–	–
WETA	0	–	–	–	–
BTYW	0/1	–	–	–	–
CAVI	0	–	–	–	–
PUFI	0	–	–	–	–
WEBL	0	–	–	–	–
WBNU	0/3	–	–	–	–
OATI	0/20	–	–	–	–
CALT	0/1	–	–	–	–
BUSH	0	–	–	–	–
LEGO	0	–	–	–	–
WAVI	0/2	–	–	–	–
PSFL	0	–	–	–	–
**Subtotal**	**0/39**	**0**	**0**	**0**	**0**
*Non-infected birds carrying ticks*					
ACWO	0	–	–	–	–
HOWR	0	–	–	–	–
HUVI	0	–	–	–	–
**Subtotal**	**0/0**	**0**	**0**	**0**	**0**
**Totals**	**25/182**	**24**	**1**	**0**	**0**

Numbers in **bold** with an asterisk (*) indicate matching genospecies between individual bird and hosted ticks, for the indicated category. Acronyms in table are: BBSL, *B*. *burgdorferi* s.l; BBSS, *B*. *burgdorferi* sensu stricto; BBIS, *B*. *bissettii*; BBSL(un), undifferentiated genospecies in the *B*. *burgdorferi* sensu lato complex that are neither BBSS nor BBIS; and BBSLmix, any combination of BBSL(un), BBSS, and BBIS detected in a single sample.

**Table 7:** Summary of *B*. *burgdorferi* s.l. genospecies found in *I*. *pacificus* nymphs removed from birds.

4 Letter species code	Raw proportion of BBSL-infected nymphs (%)	BBSL genospecies			
		BBSS	BBIS	BBSL(un)	BBSLmix
*Birds infested by infected larvae (with or without infection of the birds)*					
CATH	0/1	–	–	–	–
BUOR	0/3	–	–	–	–
SPTO	0/1	–	–	–	–
CHSP	0/0	–	–	–	–
AMRO	0/1	–	–	–	–
BEWR	1/7 (14.3)	1	–	–	–
LASP	4/14 (28.6)	3	–	–	1
OCWA	0/0	–	–	–	–
DEJU	5/14 (35.7)	1,1*	1	1	1
**Subtotal**	**10/41**	**6**	**1**	**1**	**2**
*Infected birds without infected larvae*					
RWBB	0/0	–	–	–	–
WEWP	0/0	–	–	–	–
GCSP	0/0	–	–	–	–
STJA	4/7 (57.1)	3	1	–	–
BRCR	0/8	–	–	–	–
BHGR	0/2	–	–	–	–
WETA	0/0	–	–	–	–
BTYW	0/0	–	–	–	–
CAVI	0/1	–	–	–	–
PUFI	0/1	–	–	–	–
WEBL	1/3 (33.3)	–	1	–	–
WBNU	0/0	–	–	–	–
OATI	2/14 (14.3)	1	–	1	–
CALT	0/0	–	–	–	–
BUSH	0/0	–	–	–	–
LEGO	0/0	–	–	–	–
WAVI	0/1	–	–	–	–
PSFL	0/1	–	–	–	–
**Subtotal**	**7/38**	**4**	**2**	**1**	**0**
*Non-infected birds carrying ticks*					
ACWO	1/2 (50.0)	1	–	–	–
HOWR	2/8 (25.0)	–	2	–	–
HUVI	1/1 (100.0)	1	–	–	–
**Subtotal**	**4/11**	**2**	**2**	**0**	**0**
**Totals**	**21/90**	**12**	**5**	**2**	**2**

Numbers in **bold** with an asterisk (*) indicate matching genospecies between individual bird and hosted ticks, for the indicated category. Acronyms in table are: BBSL, *B*. *burgdorferi* s.l; BBSS, *B*. *burgdorferi* sensu stricto; BBIS, *B*. *bissettii*; BBSL(un), undifferentiated genospecies in the *B*. *burgdorferi* sensu lato complex that are neither BBSS nor BBIS; and BBSLmix, any combination of BBSL(un), BBSS, and BBIS detected in a single sample.

The Lesser Goldfinch (*Spinus psaltria*), Oak Titmouse (*Baeolophus inornatus*) and Dark-eyed Junco (*Junco hyemalis*) were the only species harboring all four subcategories of BBSL infection, whereas the Bewick’s Wren (*Thryomanes bewickii*) yielded 3 subcategories (BBSS, BBIS, BBSL(un)). The American Robin and Dark-eyed Junco were the only species with infections in both individual birds and the larvae removed from them, although the genospecies differed in the birds and larvae.

Compared to the sample population, the Golden-crowned Sparrow (*Zonotrichia atricapilla*) was infected with BBSS more frequently than other species (4 of 14 infected; two-tailed P < 0.0001, Pearson’s Chi-squared test). Lark Sparrows (*Chondestes grammacus*) yielded a much higher percentage of BBSS-infected larvae removed from birds than did other bird species (14/79, 17.7%). However, these larvae were removed from two heavily infested individual birds, and therefore the results must be interpreted cautiously.

### Null model for infection and infestation: average body weight

**Fig 2:**
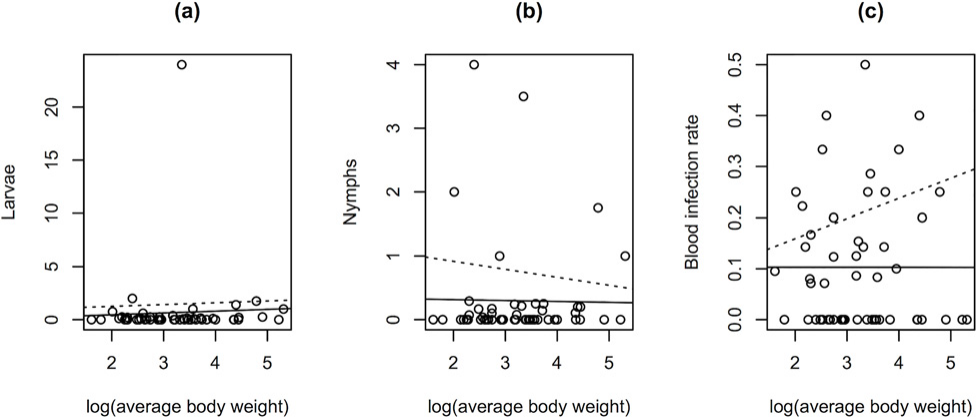
Body weight as a null model for presence or absence of tick infestation and *B*. *burgdorferi* s.l. infection in birds. Graphs depict: (a) larvae per bird by log(average body weight), (b) nymphs per bird by log(average body weight), and (c) bird infection by log(average body weight). Each data point represents one bird species. One outlier point in plot (a) represents Lark Sparrows (*Chondestes grammacus*). The solid line is a regression on all species, while the dotted-line is a regression on zero-truncated data (species with positive results only).

Table 3 summarizes the main results of all modeling goals. To test the hypotheses that larval and nymphal loads are proportional to the (logarithmically transformed) average body weight of a bird species, and that infection depends similarly on body weight, species’ average body weights were log-transformed. Single-variable linear regression models showed that average body weight of birds by species (*n* = 53) did not predict the number of larvae (F_1,50_ = 0.07912; P = 0.7797) or the number of nymphs (F_1,51_ = 0.0124; P = 0.9118) carried per bird, or BBSL infection prevalence in a given species (F_1,51_ = 3.124x10^–5^; P = 0.9956). Models constructed from positive results only, or a “zero-truncated” dataset, showed no significant correlations between body weight by species and number of larvae carried per bird (F_1,22_ = 0.0237; P = 0.8791) or number of nymphs (F_1,19_ = 0.1954; P = 0.6635) carried per bird, or BBSL infection prevalence (F_1,25_ = 2.133; P = 0.1566). See Fig. 2.

### Model exploration and selection, BBSL infection

In binomial models for BBSL infection in birds that included the orders Piciformes (woodpeckers) and Passeriformes, the presence of BBSL infection was best explained by number of nymphs removed from a bird, year, taxonomic order, and breeding status (AIC evidence ratio 2.41 between best and next best model; AIC weight = 0.55). BBSL was detected in 23 bird species in the order Passeriformes and in larvae removed from 9 species, but no members of the order Piciformes (31 individuals in 4 genera and 4 species) were infected or harbored infected larvae. In terms of *I*. *pacificus* infestation, only two nymphs were found on two separate Acorn Woodpeckers (*Melanerpes formicivorus*), and only one larva was found on one of four Northern Flickers (*Colaptes auratus*), the only ground-feeding woodpecker captured. Restricting further BBSL-infection analyses to the order Passeriformes, we find that presence of BBSL infection was best explained by number of nymphs removed from a bird, year, and breeding status (AIC evidence ratio 1.2 between best and next best model; AIC weight = 0.31).

In both the combined Passeriformes and Piciformes model, and the Passeriformes-only model, the non-breeding status of birds was highly significant. However, only 20 of the 623 sampled birds were non-breeders; 14 of these were Golden-crowned Sparrows (*Zonotrichia atricapilla*). This species contained all four infected, non-breeding birds. This indicates that the significance of non-breeding status in these models was attributable to the prevalence of infection in this one species. Re-evaluating both models without breeding status as a variable, we found that bird infection was best predicted by number of nymphs removed from a bird, year, and taxonomic order for models including both Passeriformes and Piciformes; and number of nymphs removed from a bird, year, and log (average body weight) for the Passeriformes-only model. Details of model selection, parameter estimates and significance for these and all modeling goals are available in Tables B-G in S2 File, and Tables H-I in S3 File. Raw data were graphed by variable of interest for visual comparisons and is available in Figures A-F in S1 File.

### Model selection, presence of larvae on birds

Only 62 of 623 individual birds were found to carry one or more larvae. The binomial model that was best supported by AICc values for the orders Passeriformes and Piciformes was presence of larvae on individual birds ~ year + taxonomic order + feeding substrate. The significant variables and their levels were the intercept and year (highly significant), the order Piciformes (which was negatively correlated with the presence of larvae), and in the category of feeding substrate, the levels “bark” and “ground” both showed a significant positive correlation with larval presence.

When limited to the order Passeriformes only, the best-supported model was presence of larvae on individual birds ~ year + nesting substrate + feeding substrate, where the intercept and year again showed high significance. The nesting substrates “shrub” and “tree” both showed significantly lower risk of larval infestation than other nest placement substrates (ground, bark). Bark and foliage feeding substrates were both significant and positively correlated with larval presence, and ground as a feeding substrate showed high significance for predicting larval presence. See Tables D-E in S2 File.

### Model selection, presence of nymphs on birds

We considered the presence of nymphs on the orders Passeriformes and Piciformes, and noted that only 54 of 620 birds (8.7%) had nymphs. The best-supported binomial model for nymphal presence was presence of nymphs on individual birds ~ year + taxonomic family, where the intercept was not significant, and year was highly significant. While no level of family was significant by itself, the inclusion of this variable was responsible for best model fits. Most instances of nymphs removed from birds came from the taxonomic families Certhiidae, Corvidae, and Troglodytidae, however, the observations are too limited to yield significant results. Because the number of genera was greater than the number of family included in this study, it appears that model selection was not biased in favor of the category with the greatest number of levels.

When the Passeriformes-only models were compared, the best-supported model was y ~ year + taxonomic family + main habitat, where the intercept was again not significant, and year was highly significant. Additionally, the main habitat category “dense oak woodland” showed a highly significant and positive correlation with nymphal presence. Once again, inclusion of the variable “family” yields the best-model fits, though none of the levels of family was by itself significant. See Tables F-G in S2 File.

### Model selection, counts of larvae on birds

Both the orders Passeriformes and Piciformes were considered in these analyses. Models consisted of a negative binomial count model with a log link, and a zero-inflation binomial model with a logit link, in the form y ~ (count model | zero-inflated model). For the top supported model counts of larvae on individual birds ~ (main habitat + feeding substrate | year + feeding substrate), the count model showed the following levels of main habitat to be highly significant: grass (P < 0.0001); oak woodland (P = 0.0248); dense oak woodland (P = 0.0059). The level “grass” has the largest predicted positive effect, followed by dense oak woodland, oak woodland, and finally chaparral, which was negatively correlated with larval counts. Although feeding substrate was significant, none of the levels were by themselves significant. For the zero-inflation part of the model, the intercept is highly significant (P < 0.0001). Complete model results are available in Table H in S3 File.

### Model selection, counts of nymphs on birds

These analyses also include the orders Passeriformes and Piciformes. Here, y = number of nymphs removed from an individual bird. For the top supported model, y ~ (main habitat | log (average body weight) + year), the count model variable levels predicting higher numbers of nymphs on birds were main habitat = grass (P = 0.0004), oak woodland (P = 0.0505), and dense oak woodland (P = 0.0082), than the reference level “chaparral,” again with “grass” having the largest effect, followed by dense oak woodland, and oak woodland. Intercept was also significant in the count model (P = 0.0002). Intercept and log(average body weight) were significant in predicting presence of nymphs on birds. Complete model results are available in Table I in S3 File.

## Discussion

Our findings demonstrate that in northwestern California, birds contribute to the enzootic maintenance of BBSL spirochetes, given the number of bird species (32/53) and individual birds (100/623) hosting *I*. *pacificus*, and the prevalence of BBSL both in birds and in larvae removed from them. Our results also demonstrate that in addition to taxonomic relatedness, certain bird life history traits predict BBSL infection. In contrast, null models based on bird body weight by species as predictive models of BBSL infection, larval loads, and nymphal loads on birds did not explain any of these phenomena. Models that performed best were those that incorporated bird behaviors, taxonomic relationships, and sampling year. With regard to our original hypotheses, we found:

(1) As expected, the number of infesting nymphs was highly significant in predicting bird infection with BBSL;
(2) Counter to expectation, average body weight by species did not serve as an adequate null model of infestation and infection prevalence;
(3) Although ground-feeding behavior was a highly significant predictor of larval presence (P = 0.0009 among passerines) in agreement with Weisbrod and Johnson [22] and Wright et al. [17], [19], contact with the ground through nest placement was not a significant factor in any of the models we tested. Our results support previously published findings indicating a much higher prevalence of infestation among ground-foraging birds [16,22,26,59], but did not support the hypothesis that ground-nesting *per se* is an important factor in predicting infestation, as implied in other studies [17,19,22];
(4) As expected, contact with bark while feeding significantly explained larval presence on individual birds in binomial models (in contrast to the findings of [19]), but use of bark as a nesting substrate did not significantly predict either tick-infestation prevalence or bird infection prevalence;
(5) Counter to our hypothesis, non-breeding birds manifested a higher level of BBSL infection (25%) compared to breeding birds (11.5%) (Passeriformes only). This result was entirely due to high levels of infection among Golden-crowned Sparrows, a non-resident species, which led us to abandon breeding status as a testable variable. Although it may be that infection is more prevalent among breeding or non-breeding birds, we were not able to test that hypothesis with our limited sample size;
(6) As hypothesized, the main food item of birds did not explain larval or nymphal infestation;
(7) As hypothesized, resident status was not significant in infection or infestation models;
(8) As hypothesized, sampling year was a highly significant variable for bird infection, presence of larvae and nymphs, and the number of ticks (both larvae and nymphs) a bird carried;
(9) Although main habitat was not by itself a significant predictor of infection in birds, the sub-category (or level) “dense oak woodland” explained presence of nymphs in binomial (presence-absence) models, and was highly significant in explaining counts of larvae removed from birds in zero-inflated negative binomial models, as expected. “Grass” as habitat type was also highly significant as a predictor of the number of larvae removed from a bird (at least in part due to the presence of one heavily infested Lark Sparrow outlier in the dataset), and “oak woodland” was also significant. In contrast, the reference level “chaparral” correlated with the lowest counts of larvae and nymphs on birds of all habitat types considered;
(10) As hypothesized, best-performing models for infection and infestation contained variables representing taxonomic structure at the level of order and below. Our results suggest that the order Piciformes is unlikely to contain major reservoirs of *B*. *burgdorferi*.

Although birds contribute to the ecology of BBSL in our study system, in general, their role is likely to be ecosystem-specific, and therefore more complex than that of the primary rodent-reservoir hosts. The large number of bird species in one location, each with generally unknown reservoir competency, greatly increases the difficulty of community-level modeling [68]. Other inherent difficulties in evaluating the importance of birds in BBSL ecology is that the species make-up of the community [69] and their true abundances are notoriously difficult to estimate [70–72].

### Prevalence of BBSL genospecies in birds

*Borrelia bissettii* (BBIS) had not been reported in birds before. This spirochete was, in fact, the most prevalent genospecies detected in birds in this study (40.4%), followed by BBSS (29.8%). In contrast, most infected larvae removed from birds instead carried BBSS (96.0%, versus 4.0% for BBIS). This discrepancy may have occurred because of the uneven abundance distribution of larvae across individual birds (leading to biased sampling), but also suggests the possibilities of a differential rate of transmission of BBSL genospecies from birds to *I*. *pacificus* larvae, or more transient infection of birds with BBIS. As we only sampled bird blood, we cannot exclude the possibility that BBSS is present in surface tissues (e.g., skin) more often than BBIS, and is therefore more easily transmitted to feeding ticks. Similarly, infected *I*. *pacificus* nymphs predominantly contained BBSS (57.1%, versus 23.8% for BBIS). Co-feeding infection, in which larvae become infected via feeding in close proximity to infected nymphs on a non-infected bird, might account for some infected larvae. Mixed and uncategorized *Borrelia* infections were present at much lower levels in birds or ticks removed from them.

Previously, BBIS had only been found in various species of mammals in North America (reviewed in [49]). Although nearly all cases of Lyme disease in North America are attributed to *B*. *burgdorferi*, BBIS occasionally infects people in central and southern Europe [2], and a genospecies closely related to, if not identical with, BBIS was detected in serum specimens from three persons residing in northwestern California [47].

### Suggestions for future studies

Four aspects of our data suggest important considerations in the design of future studies. First, no birds carried the same genospecies of BBSL in both their blood and in larvae feeding on them. Of 12 individual birds (in 9 species) that carried infected larvae, birds either had a different genospecies of BBSL in their blood (3 individuals in 2 species) or tested negative for blood infection (9 individuals in 8 species; Tables 4–7). Investigations of skin, feather attachments, and other surface tissues of birds may be useful in discovering the tissue tropisms of BBSL in birds [73]. Considering the skin-tropisms of certain Lyme disease spirochetes in mammals, and that cultures are often obtained from ear-punch biopsies from rodents, examination of more types of bird tissues may result in (non-lethal) field-sampling techniques that generate a higher rate of positive test results among infected animals [19,74].

Second, certain bird species’ immune systems may clear BBSL infections from their blood. If so, and the feeding attraction of infection-cleared hosts is of sufficient magnitude, these species would constitute “dilution hosts” of BBSL by reducing the overall prevalence of spirochetes in the vector-tick population. Clearance, or marked suppression, of infection could also lead to the observation cited in the previous paragraph: infected, blood-feeding larvae on birds lacking blood infection may signify transient bacteremia in those birds. Investigations of host competency of birds by species, and mechanisms by which birds clear infection from their blood are needed. For example, blood pH-raising effects of high-altitude migrations [75] may affect the abundance of *Borrelia* spirochetes in bird blood, and blood-temperature increases induced by migration and periods of high activity [76] may exceed the tolerance range of some *Borrelia* genospecies [77]. Additionally, the age class of birds, unmeasured in this study, may correlate with naturally acquired immunity.

Third, attempts to model the dynamics of BBSL infections in wild bird populations will require assessments of the true species diversity and estimations of true abundances by species within each habitat type, including birds that are potential hosts but are not adequately sampled with mist-netting techniques. Mist netting, compared to other bird survey techniques, can give highly biased estimates of bird abundances and species composition by excluding birds that do not fly at the level of the mist net or are otherwise excluded from this survey technique, such as large birds, nocturnal birds and ground-dwelling birds [78,79]. With true bird abundance information obtained through a variety of methods such as point counts and trapping, total tick loads and relative importance of each host species could be estimated from tick data obtained from caught birds.

Of the 219 bird species observed in the Hopland study area, many species known to be residents or breeders in the ecosystems studied were either underrepresented or not represented in our dataset [54]. These include the American Robin, which is a competent reservoir host for *B*. *burgdorferi* in the northeastern United States [29]. Although American Robins are common to abundant during all seasons at the HREC, only five birds were collected during this study. Similarly, two abundant species of quail at the HREC yielded only 1 individual bird to our mist netting and ground-trapping efforts, and the fairly common Wild Turkey (*Meleagris gallopavo*) was not captured at all [20]. Among raptors (hawks, falcons, eagles, vultures, and owls), 1 of 22 species occurring there was captured during the present study. Such predatory and scavenging birds, especially those that feed primarily on mammals, may be attractive to partially fed nymphs that detach from their dead hosts [80]. Capture methods appropriate to the life histories of each species of birds may increase the diversity of birds that can be sampled [81]. A more complete understanding of the entire bird community and their relative abundances may reveal additional important reservoirs of BBSL.

Lastly, landscape dynamics are particularly critical to understand in California, where losses of chaparral and oak woodland ecosystems due to fragmentation, removal, and other anthropogenically-induced changes are causing biodiversity loss and extreme changes to the existing bird communities [82–87]. Chaparral yielded the lowest prevalence of larval and nymphs ticks on birds at the HREC, although recent studies targeting rodents and lizards at the there [62,88] demonstrated abundant subadult *I*. *pacificus* larval and nymphal populations in this habitat type. Rapid shifts in California bird community structures towards locally increased abundances of some known carriers of *Borrelia* spirochetes (e.g. American Robins and Dark-eyed Juncos) as a result of urbanization [89] and chaparral removal in northwest California (Newman et al., *in preparation*) represents changes in local disease ecology dynamics [90] and the potential for increased transmission of Lyme disease spirochetes to humans.

## Supporting Information

S1 File (Table A, Figure A, Figure B, Figure C, Figure D, Figure E, Figure F). **Table A.** Information on body weight and ecological guilds for all species of birds included in this study. Categories of analysis, their levels, and level abbreviations are shown in the main text in Table 2. **Figures A-F.** Boxplots of raw data larval infestation, nymphal infestation, and bird infection status by variable of interest.
(DOCX)

S2 File (Tables B, Tables C, Tables D, Tables E, Tables F, Tables G). Presence models, model selection AIC values, and parameter estimates for binomial models of bird infection, larval infestation, and nymphal infestation of birds. **Table B.** Model selection second-order Akaike Information Criterion (AICc) values for binomial models of BBSL **blood infection** in birds (and in larvae removed from those birds). Models include factors specified in the main article and bird traits including the orders Passeriformes and Piciformes. **Table C.** Model selection second-order Akaike Information Criterion (AICc) values and approximate P-values for variables in the top selected model. The models below are binomial models of BBSL **blood infection** in birds (and in larvae removed from those birds). Models include factors specified in the main article and bird traits limited to the order Passeriformes. **Table D.** Model selection second-order Akaike Information Criterion (AICc) values for bird life history traits that lead to *Ixodes pacificus* **larvae presence** on a bird, including the orders Passeriformes and Piciformes. **Table E.** Model selection on second-order Akaike Information Criterion (AICc) values for binomial models of bird life history traits and sampling conditions that lead to *I*. *pacificus* **larvae presence** on a bird, limited to the order Passeriformes. **Table F.** Model selection for bird traits and sampling conditions that lead to *Ixodes pacificus* **nymph presence** on a bird, including the orders Passeriformes and Piciformes. **Table G.** Model selection for bird traits and sampling conditions that result in *Ixodes pacificus* **nymph presence** on a bird, limited to the order Passeriformes.
(DOCX)

S3 File (Tables H, Tables I). Count models, model selection AIC values, and parameter estimates for zero-inflated negative binomial models of larval and nymphal infestations of birds **Tables H.** Model selection AIC values for bird life history traits that predict **number of *Ixodes pacificus* larvae removed from a bird**, including the orders Passeriformes and Piciformes **Tables I.** Model selection AIC values for bird life history traits that lead to **number of *Ixodes pacificus* nymphs removed from a bird**, including the orders Passeriformes and Piciformes
(DOCX)
