# Supplementary material for: Borrelia burgdorferi Sensu Lato Spirochetes in Wild Birds in Northwestern California: Associations with Ecological Factors, Bird Behavior and Tick Infestation
Source: PLoS One. 2015 Feb 25;10(2):e0118146. doi: 10.1371/journal.pone.0118146 (PMC4340631; doi:10.1371/journal.pone.0118146)

**Supporting Information**

**File S1.** Information on body weight and ecological guilds for all species of birds included in this study. Categories of analysis, their levels, and level abbreviations are shown in the main text in Table 2.

**Table A.** Information on body weight and ecological guilds for all species of birds included in this study. Categories of analysis, their levels, and level abbreviations are shown in the main text in Table 2.

| **Common name** | **4-letter code** | **Average body weight (in grams)** | **Breeding status** | **Feeding substrate** | **Nesting substrate** | **Main food item** | **Main habitat** |
| --- | --- | --- | --- | --- | --- | --- | --- |
| Acorn Woodpecker | ACWO | 77.5 | BREED | BARK | CAVITY | INSECTS | OAKW |
| American Robin | AMRO | 81.0 | BREED | GROUND | TREE | INSECTS | OAKW |
| Ash-throated Flycatcher | ATFL | 29.5 | BREED | HOVERING | CAVITY | INSECTS | OAKW |
| Bewick's Wren | BEWR | 10.0 | BREED | FOLIAGE | CAVITY | INSECTS | OAKW |
| Blue-gray Gnatcatcher | BGGN | 6.0 | BREED | FOLIAGE | TREE | INSECTS | XW |
| Brown-headed Cowbird | BHCO | 46.0 | BREED | GROUND | OPP | SEEDS | GRASS |
| Black-headed Grosbeak | BHGR | 42.0 | BREED | FOLIAGE | TREE | INSECTS | XW |
| Black Phoebe | BLPH | 18.5 | BREED | FLYCATCHING | CLIFF | INSECTS | OAKW |
| Brown Creeper | BRCR | 7.5 | BREED | BARK | BARK | INSECTS | XW |
| Black-throated Gray Warbler | BTYW | 8.5 | BREED | FOLIAGE | TREE | INSECTS | XW |
| Bullock's Oriole | BUOR | 36.0 | BREED | FOLIAGE | TREE | INSECTS | OAKW |
| Bushtit | BUSH | 5.0 | BREED | FOLIAGE | TREE | INSECTS | CHAP |
| California Towhee | CALT | 52.0 | BREED | GROUND | SHRUB | SEEDS | CHAP |
| California Quail | CAQU | 185.0 | BREED | GROUND | GROUND | SEEDS | CHAP |
| California Thrasher | CATH | 85.5 | BREED | GROUND | SHRUB | INSECTS | CHAP |
| Cassin's Vireo | CAVI | 15.5 | BREED | FOLIAGE | TREE | INSECTS | XW |
| Chipping Sparrow | CHSP | 13.5 | BREED | GROUND | SHRUB | SEEDS | OAKW |
| Dark-eyed Junco | DEJU | 24.0 | BREED | GROUND | GROUND | SEEDS | XW |
| Downy Woodpecker | DOWO | 24.5 | BREED | BARK | CAVITY | INSECTS | XW |
| European Starling | EUST | 78.0 | BREED | GROUND | CAVITY | INSECTS | GRASS |
| Fox Sparrow | FOSP | 35.3 | NON | GROUND | GROUND | INSECTS | XW |
| Golden-crowned Sparrow | GCSP | 31.5 | NON | GROUND | GROUND | INSECTS | CHAP |
| House Wren | HOWR | 11.0 | BREED | FOLIAGE | CAVITY | INSECTS | OAKW |
| Hutton's Vireo | HUVI | 12.0 | BREED | FOLIAGE | TREE | INSECTS | XW |
| Lark Sparrow | LASP | 28.5 | BREED | GROUND | GROUND | SEEDS | GRASS |
| Lazuli Bunting | LAZB | 15.5 | BREED | GROUND | SHRUB | INSECTS | OAKW |
| Lesser Goldfinch | LEGO | 9.8 | BREED | FOLIAGE | TREE | SEEDS | OAKW |
| Lincoln's Sparrow | LISP | 18.0 | NON | GROUND | GROUND | INSECTS | CHAP |
| Nashville Warbler | NAWA | 9.5 | NON | FOLIAGE | GROUND | INSECTS | XW |
| Northern Flicker | NOFL | 135.0 | BREED | GROUND | CAVITY | INSECTS | OAKW |
| Nuttall's Woodpecker | NUWO | 37.5 | BREED | BARK | CAVITY | INSECTS | OAKW |
| Oak Titmouse | OATI | 15.5 | BREED | FOLIAGE | CAVITY | INSECTS | OAKW |
| Orange-crowned Warbler | OCWA | 9.0 | BREED | FOLIAGE | GROUND | INSECTS | XW |
| Pacific-slope Flycatcher | PSFL | 10.0 | BREED | FLYCATCHING | CAVITY | INSECTS | XW |
| Purple Finch | PUFI | 25.0 | BREED | FOLIAGE | TREE | SEEDS | XW |
| Rufous-crowned Sparrow | RCSP | 19.3 | BREED | GROUND | GROUND | SEEDS | CHAP |
| Red-eyed Vireo | REVI | 19.0 | NON | FOLIAGE | TREE | INSECTS | N/A |
| Red-winged blackbird | RWBB | 54.5 | BREED | GROUND | SHRUB | INSECTS | GRASS |
| Sage Sparrow | SAGS | 18.5 | BREED | GROUND | SHRUB | INSECTS | CHAP |
| Song Sparrow | SOSP | 32.5 | BREED | GROUND | SHRUB | INSECTS | OAKW |
| Spotted Towhee | SPTO | 41.0 | BREED | GROUND | GROUND | OMNIVORE | CHAP |
| Steller's Jay | STJA | 120.0 | BREED | GROUND | TREE | OMNIVORE | XW |
| Swainson's Thrush | SWTH | 34.0 | NON | FOLIAGE | SHRUB | INSECTS | XW |
| Violet-green Swallow | VGSW | 14.0 | BREED | AERIAL | CAVITY | INSECTS | OAKW |
| Warbling Vireo | WAVI | 13.0 | BREED | FOLIAGE | TREE | INSECTS | OAKW |
| White-breasted Nuthatch | WBNU | 24.0 | BREED | BARK | CAVITY | INSECTS | XW |
| Western Bluebird | WEBL | 27.5 | BREED | FLYCATCHING | CAVITY | INSECTS | OAKW |
| Western Scrub-Jay | WESJ | 85.0 | BREED | GROUND | TREE | OMNIVORE | CHAP |
| Western Screech-Owl | WESO | 202.5 | BREED | STALKING | CAVITY | MAMMALS | OAKW |
| Western Tanager | WETA | 30.0 | BREED | FOLIAGE | TREE | INSECTS | XW |
| Western Wood-Pewee | WEWP | 12.5 | BREED | FLYCATCHING | TREE | INSECTS | OAKW |
| Wrentit | WREN | 14.5 | BREED | FOLIAGE | SHRUB | INSECTS | CHAP |
| Yellow-rumped Warbler | YRWA | 12.5 | BREED | FOLIAGE | TREE | INSECTS | XW |

**Figures A-F.** Boxplots of raw data larval infestation, nymphal infestation, and bird infection status by variable of interest.

For the following graphs, upper and lower whiskers are defaults within R “plot” command, in the “graphics” package version 3.0.1 (Murrell, P. [2005] *R Graphics*. Chapman & Hall/CRC Press).

**Figure A.** Infestation and bird infection by breeding status


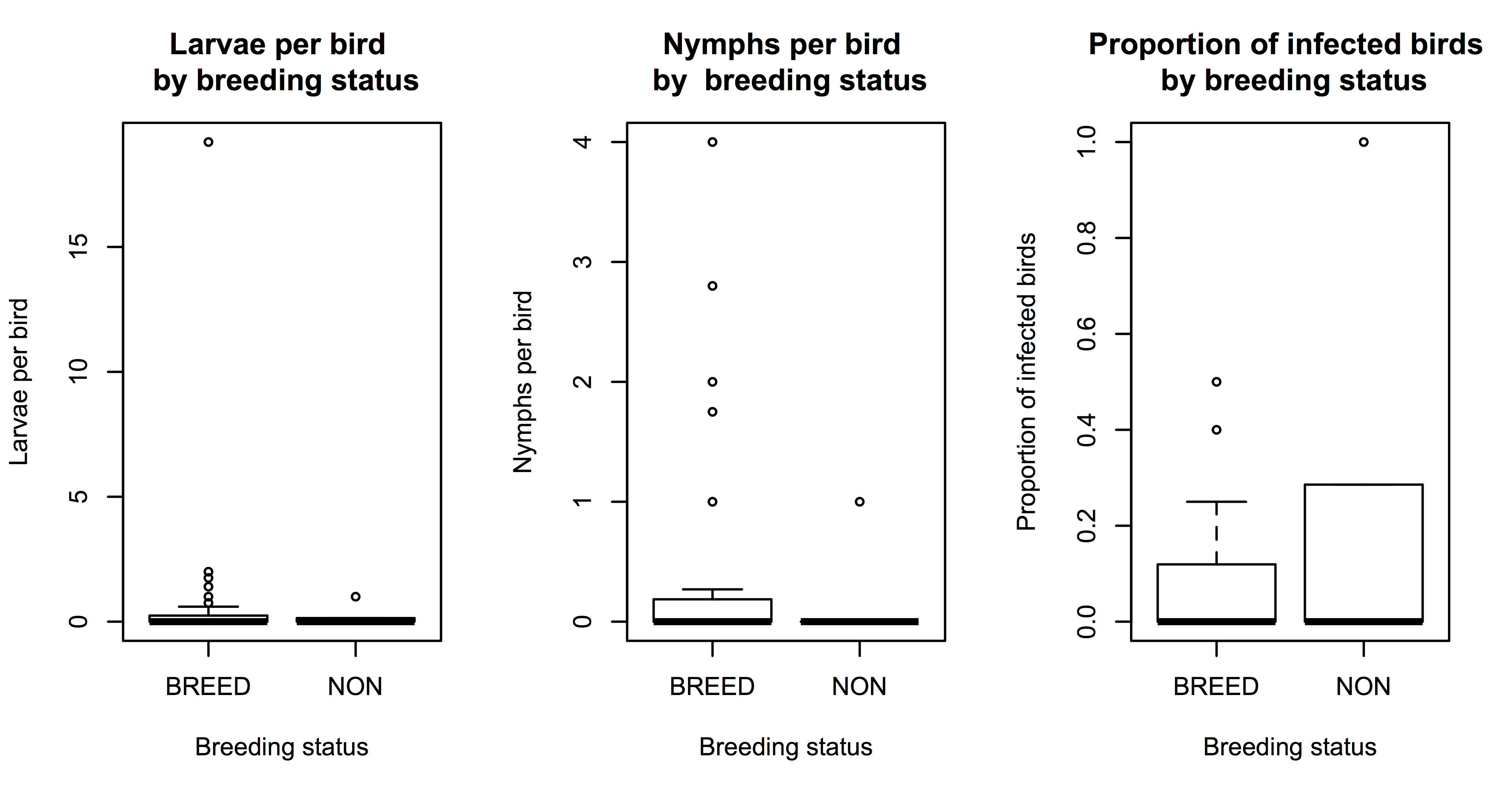


**Figure B.** Infestation and bird infection by feeding substrate

**
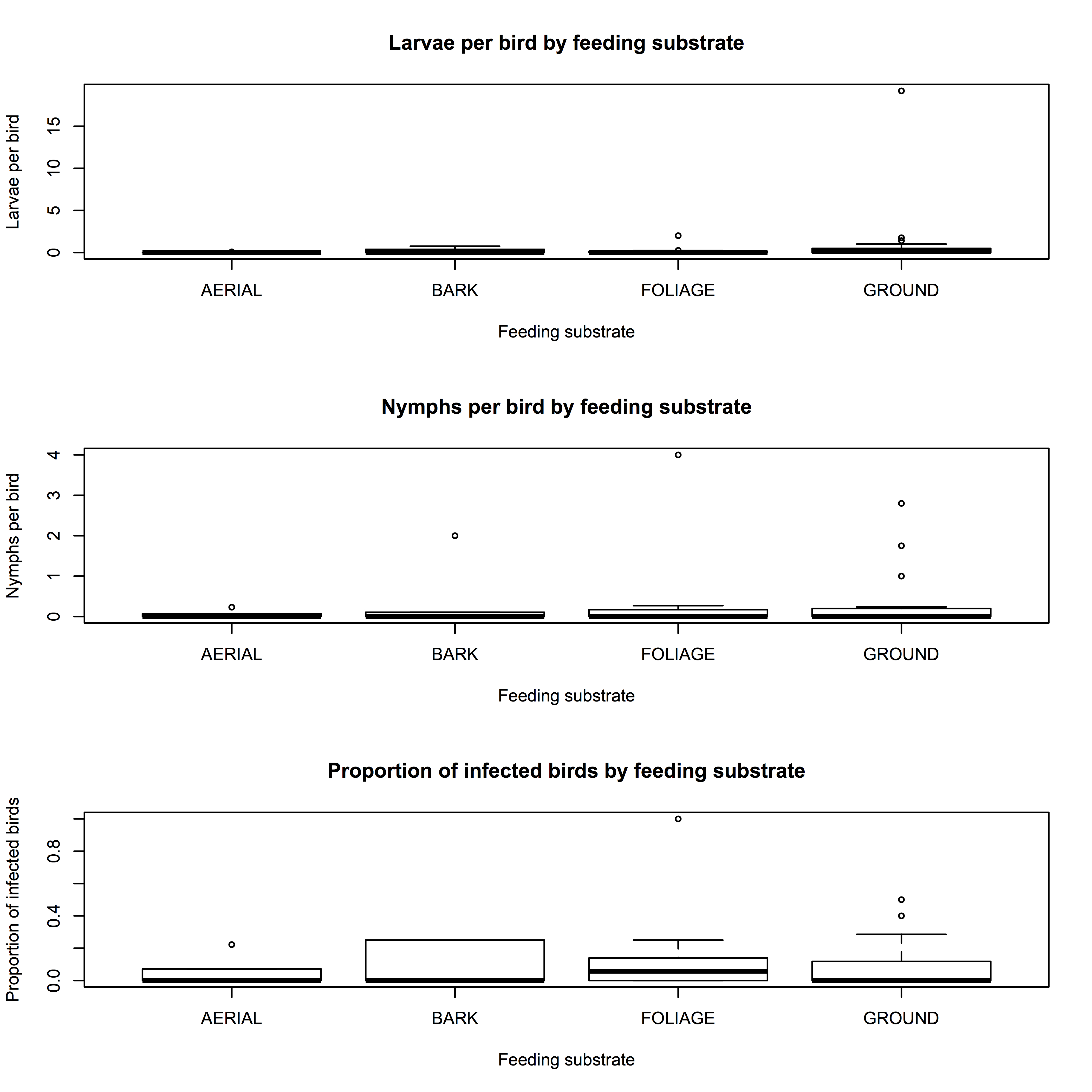
**

**Figure C.** Infestation and bird infection by main food item

**
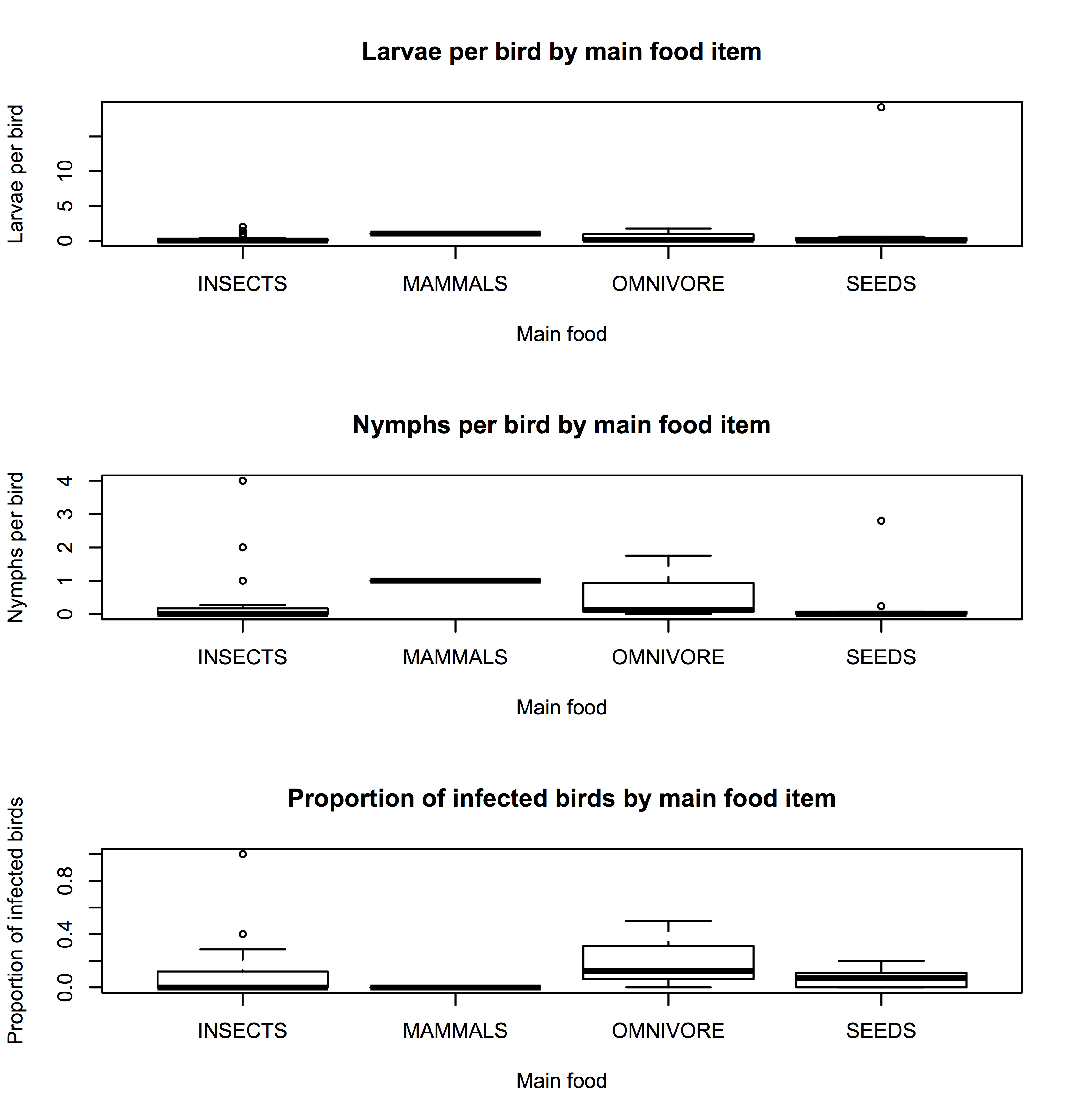
**

**Figure D.** Infestation and bird infection by main habitat

**
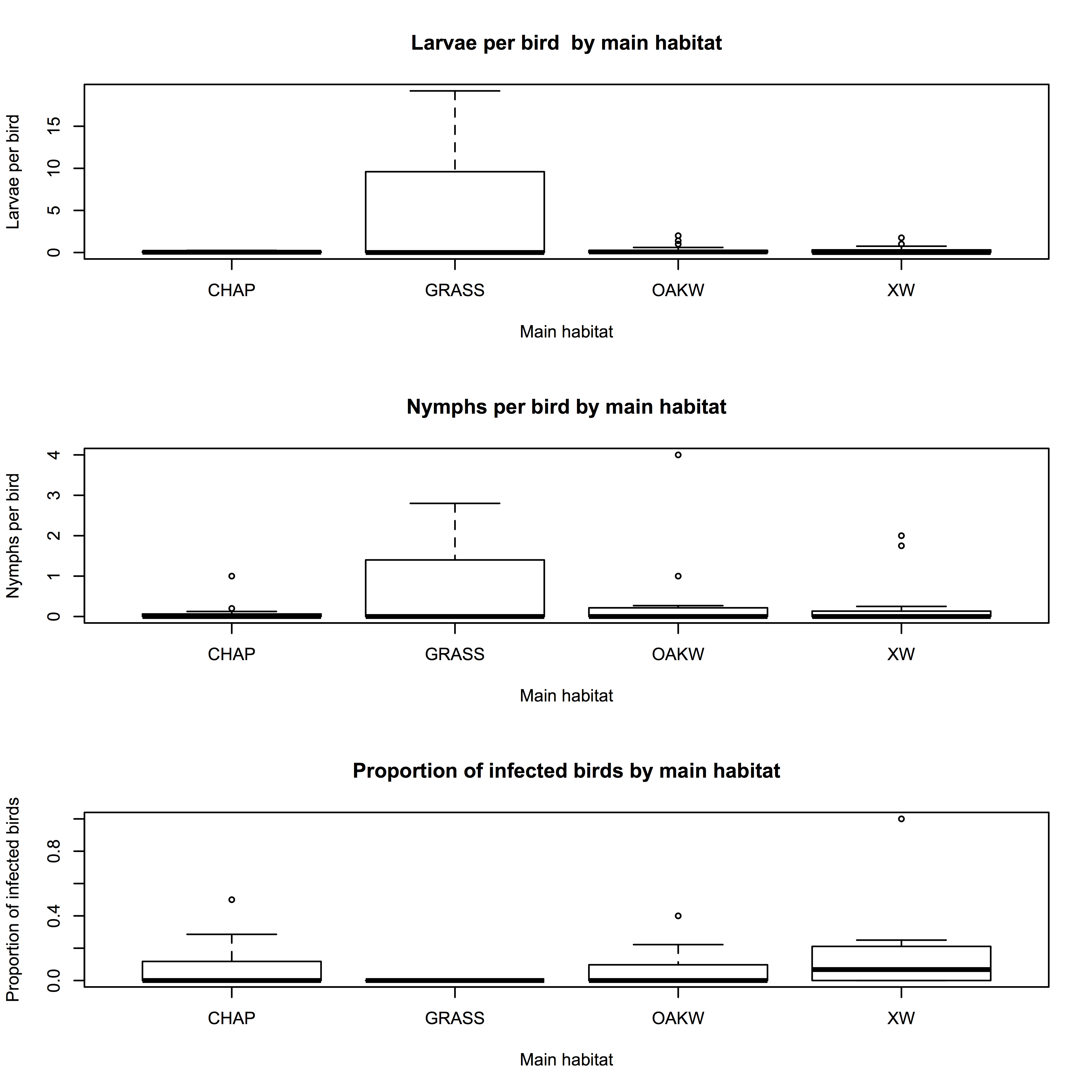
**

**Figure E.**

**
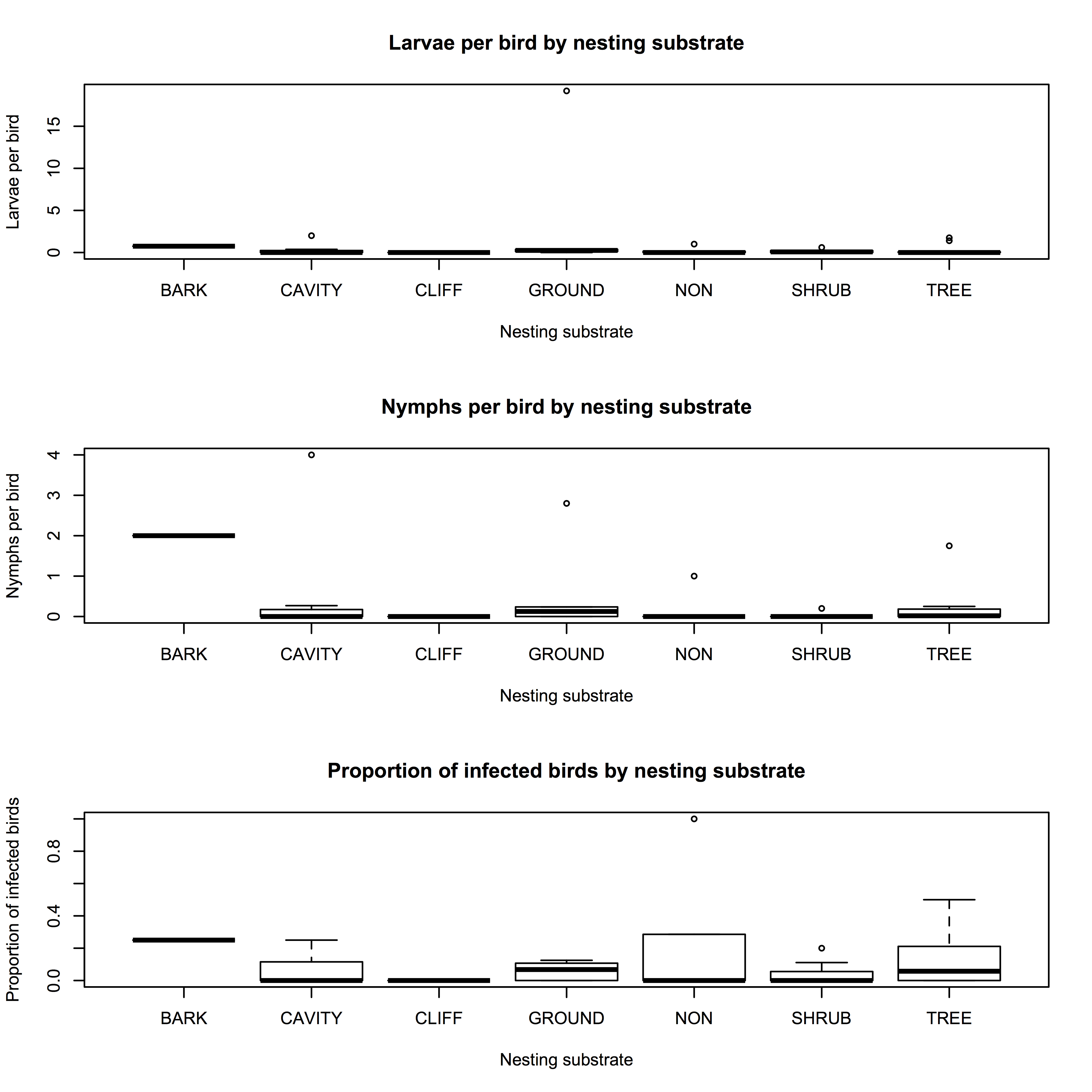
**

**Figure F.** Infestation and bird infection by resident status


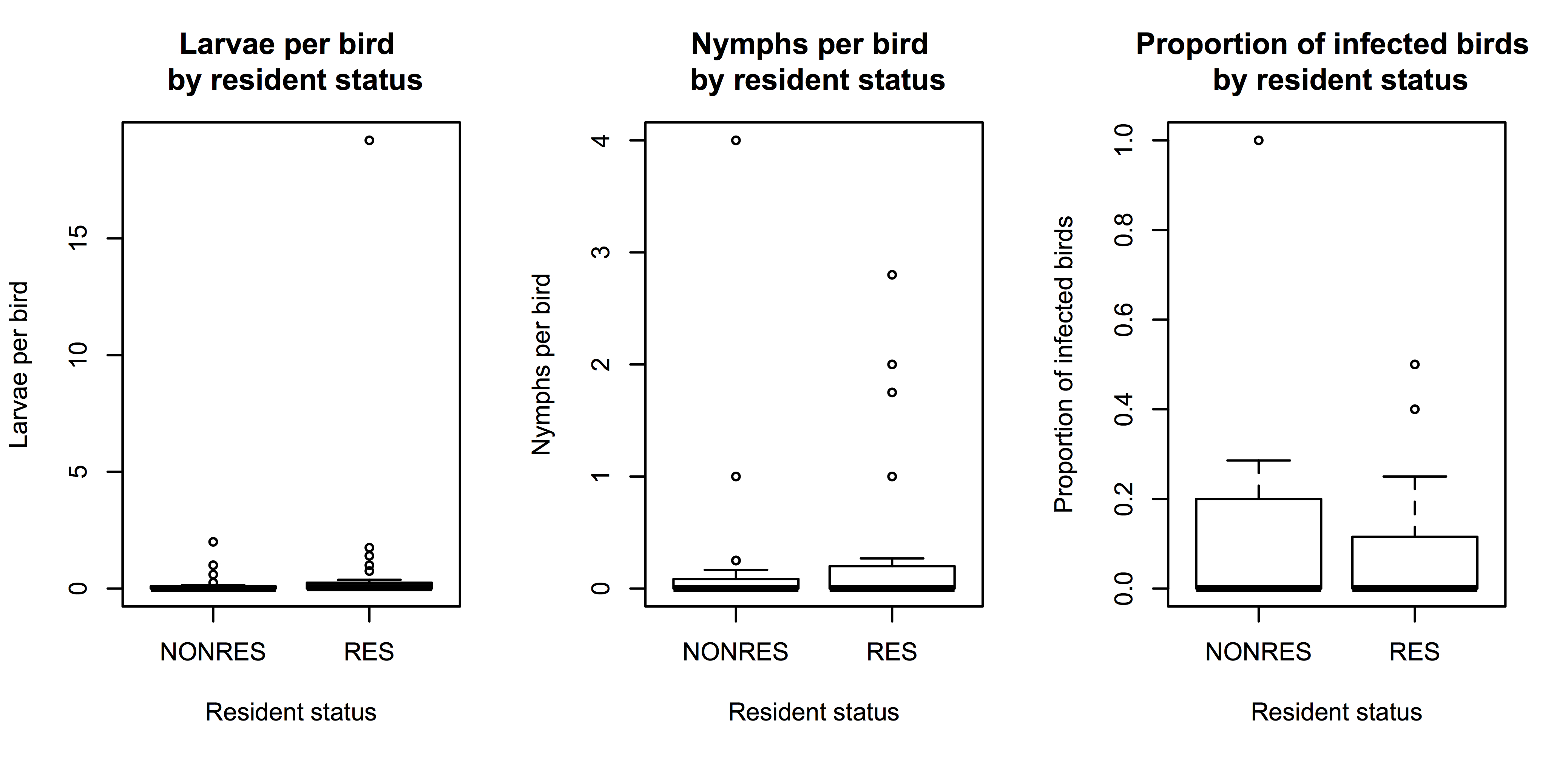

Supplement: S1 File — Table A. Information on body weight and ecological guilds for all species of birds included in this study. Categories of analysis, their levels, and level abbreviations are shown in the main text in Table 2. Figures A-F. Boxplots of raw data larval infestation, nymphal infestation, and bird infection status by variable of interest. (DOCX) [file pone.0118146.s001.docx]
