# Supplementary material for: Borrelia burgdorferi Sensu Lato Spirochetes in Wild Birds in Northwestern California: Associations with Ecological Factors, Bird Behavior and Tick Infestation
Source: PLoS One. 2015 Feb 25;10(2):e0118146. doi: 10.1371/journal.pone.0118146 (PMC4340631; doi:10.1371/journal.pone.0118146)
Supplement: S2 File — Presence models, model selection AIC values, and parameter estimates for binomial models of bird infection, larval infestation, and nymphal infestation of birds. Table B. Model selection second-order Akaike Information Criterion (AICc) values for binomial models of BBSL blood infection in birds (and in larvae removed from those birds). Models include factors specified in the main article and bird traits including the orders Passeriformes and Piciformes. Table C. Model selection second-order Akaike Information Criterion (AICc) values and approximate P-values for variables in the top selected model. The models below are binomial models of BBSL blood infection in birds (and in larvae removed from those birds). Models include factors specified in the main article and bird traits limited to the order Passeriformes. Table D. Model selection second-order Akaike Information Criterion (AICc) values for bird life history traits that lead to Ixodes pacificus larvae presence on a bird, including the orders Passeriformes and Piciformes. Table E. Model selection on second-order Akaike Information Criterion (AICc) values for binomial models of bird life history traits and sampling conditions that lead to I. pacificus larvae presence on a bird, limited to the order Passeriformes. Table F. Model selection for bird traits and sampling conditions that lead to Ixodes pacificus nymph presence on a bird, including the orders Passeriformes and Piciformes. Table G. Model selection for bird traits and sampling conditions that result in Ixodes pacificus nymph presence on a bird, limited to the order Passeriformes. (DOCX) [file pone.0118146.s002.docx]

**Supporting Information**

**File S2.** Presence models, model selection AIC values, and parameter estimates for binomial models of bird infection, larval infestation, and nymphal infestation of birds.

For all binomial models, the dispersion parameter = 1. Below, YEAR is treated as an integer value and its estimate represents the difference between YEAR = 2004 and reference year 2003. For interpretation of coefficients, a one unit increase in the variable and level being considered result in an effect on the response variable that is the coefficient estimate. For example, in the model estimated in Table B below, an increase of one in the number of nymphs on a bird would result in an expected 0.3456 increase in blood-infection prevalence in the sample population. Various models containing non-significant variables and many non-significant models are not shown.

**Tables B.** Model selection second-order Akaike Information Criterion (AICc) values for binomial models of BBSL **blood infection** in birds (and in larvae removed from those birds). Models include factors specified in the main article and bird traits including the orders Passeriformes and Piciformes.

| **Model** | **K** | **AICc** | **Δ AICc** | **AICcWt** | **Cum.Wt** | **LL** |
| --- | --- | --- | --- | --- | --- | --- |
| y ~ N_NYM + YEAR + ORDER + BRD | 5 | 397.46 | 0.00 | 0.55 | 0.55 | -193.68 |
| y ~ N_NYM + YEAR + ORDER | 4 | 399.21 | 1.76 | 0.23 | 0.78 | -195.57 |
| y ~ N_NYM + YEAR + BRD | 4 | 401.79 | 4.33 | 0.06 | 0.84 | -196.86 |
| y ~ N_NYM + YEAR + ORDER + FDSUB + RESSTAT | 8 | 402.30 | 4.84 | 0.05 | 0.89 | -193.03 |
| y ~ N_NYM + YEAR + ORDER + FDSUB | 7 | 402.60 | 5.14 | 0.04 | 0.93 | -194.21 |
| y ~ N_NYM + YEAR + BRD + RESSTAT | 5 | 403.35 | 5.89 | 0.03 | 0.96 | -196.62 |
| y ~ N_NYM + YEAR | 3 | 403.87 | 6.42 | 0.02 | 0.98 | -198.92 |
| y ~ N_NYM + YEAR + BRD + RESSTAT + FDSUB | 8 | 405.49 | 8.03 | 0.01 | 0.99 | -194.63 |
| y ~ N_NYM + YEAR + ORDER + NEST | 11 | 406.57 | 9.12 | 0.01 | 1.00 | -192.07 |
| y ~ YEAR | 2 | 408.40 | 10.94 | 0.00 | 1.00 | -202.19 |

Evidence ratio between top model and next best-supported model: 2.41

For the best-supported model without breeding status: glm(formula = y ~ N_NYM + YEAR + ORDER, family = binomial) (where y = c[infected, not infected]), results are summarized below:

**Estimate Std. Error z value Pr(>|z|)**

(Intercept) 2133.9561 595.4121 3.584 0.000338 ***

N_NYM 0.3456 0.1335 2.589 0.009622 **

YEAR -1.0663 0.2972 -3.587 0.000334 ***

ORDERPiciformes -15.3899 694.5259 -0.022 0.982321

---

Significance codes: 0 ‘***’ 0.001 ‘**’ 0.01 ‘*’ 0.05 ‘.’ 0.1 ‘ ’ 1

Null deviance: 420.40 on 619 degrees of freedom

Residual deviance: 391.15 on 616 degrees of freedom

Reference levels are YEAR = 2003 and ORDER = Passeriformes. Intercept, year, and number of nymphs removed from birds are highly significant predictors of blood infection in birds and the larvae removed from them. Order is significant to the model, but not significant as a variable within the top-supported model (which suggests that it explains some of the data variance, but the sample size is not large enough to confer significance).

**Tables C.** Model selection second-order Akaike Information Criterion (AICc) values and approximate P-values for variables in the top selected model. The models below are binomial models of BBSL **blood infection** in birds (and in larvae removed from those birds). Models include factors specified in the main article and bird traits limited to the order Passeriformes.

| **Model** | **K** | **AICc** | **Δ AICc** | **AICcWt** | **Cum.Wt** | **LL** |
| --- | --- | --- | --- | --- | --- | --- |
| y ~ N_NYM + YEAR + LOG.AVEBWT | 4 | 396.95 | 0.00 | 0.33 | 0.33 | -194.44 |
| y ~ N_NYM + YEAR | 3 | 397.19 | 0.24 | 0.29 | 0.63 | -195.57 |
| y ~ N_NYM + YEAR + RESSTAT | 4 | 398.33 | 1.38 | 0.17 | 0.79 | -195.13 |
| y ~ N_NYM + YEAR + RESSTAT + FDSUB | 7 | 400.26 | 3.31 | 0.06 | 0.86 | -193.03 |
| y ~ N_NYM + YEAR + FDSUB | 6 | 400.56 | 3.61 | 0.05 | 0.91 | -194.21 |
| y ~ YEAR | 2 | 401.35 | 4.40 | 0.04 | 0.95 | -198.67 |
| y ~ N_NYM + YEAR + LOG.AVEBWT + FDSUB | 7 | 401.59 | 4.64 | 0.03 | 0.98 | -193.70 |
| y ~ N_NYM + YEAR + NEST + LOG.AVEBWT | 11 | 403.80 | 6.85 | 0.01 | 0.99 | -190.67 |
| y ~ N_NYM + YEAR + NEST | 10 | 404.52 | 7.57 | 0.01 | 1.00 | -192.07 |

Evidence ratio between top model and next best-supported model: 1.13

For the best-supported model: glm(formula = y ~ N_NYM + YEAR + LOG.AVEBWT, family = binomial) (where y = c[infected, not infected]), results are summarized below:

**Estimate Std. Error z value Pr(>|z|)**

(Intercept) 2190.4919 597.2141 3.668 0.000245 ***

N_NYM 0.3242 0.1350 2.401 0.016336 *

YEAR -1.0949 0.2981 -3.673 0.000240 ***

LOG.AVEBWT 0.7384 0.4854 1.521 0.128246

---

Significance codes: 0 ‘***’ 0.001 ‘**’ 0.01 ‘*’ 0.05 ‘.’ 0.1 ‘ ’ 1

Null deviance: 413.23 on 588 degrees of freedom

Residual deviance: 388.88 on 585 degrees of freedom

Reference level is YEAR = 2003. Intercept, year, and number of nymphs removed from birds are highly significant predictors of blood infection in birds and the larvae removed from them. The logarithm of average bodyweight of individual birds is significant to the model, but not significant as a variable within the top-supported model.

**Tables D.**  Model selection second-order Akaike Information Criterion (AICc) values for bird life history traits that lead to *Ixodes pacificus* **larvae presence** on a bird, including the orders Passeriformes and Piciformes.

| **Model** | **K** | **AICc** | **Δ AICc** | **AICcWt** | **Cum.Wt** | **LL** |
| --- | --- | --- | --- | --- | --- | --- |
| y ~ YEAR + ORDER + FDSUB | 6 | 345.27 | 0.00 | 0.54 | 0.54 | -166.57 |
| y ~ YEAR + ORDER + FDSUB + RESSTAT | 7 | 346.57 | 1.29 | 0.28 | 0.82 | -166.19 |
| y ~ YEAR + MNFD + FDSUB | 7 | 348.74 | 3.47 | 0.09 | 0.92 | -167.28 |
| y ~ YEAR + RESSTAT + FDSUB | 6 | 349.55 | 4.28 | 0.06 | 0.98 | -168.71 |
| y ~ YEAR + MNHAB + FDSUB | 9 | 352.09 | 6.82 | 0.02 | 1.00 | -166.90 |
| y ~ YEAR + NEST | 9 | 356.83 | 11.56 | 0.00 | 1.00 | -169.27 |
| y ~ YEAR + ORDER + NEST | 10 | 357.11 | 11.84 | 0.00 | 1.00 | -168.38 |

Evidence ratio between top model and next best-supported model: 1.91

For the best-supported model: glm(formula = y ~ YEAR + ORDER + FDSUB, family = binomial) (where y = c[with larvae, without larvae]), model results are summarized below:

**Estimate Std. Error z value Pr(>|z|)**

(Intercept) 2882.2182 666.5862 4.324 1.53e-05 ***

YEAR -1.4408 0.3328 -4.330 1.49e-05 ***

FDSUBBARK 2.8797 1.2006 2.399 0.01646 *

FDSUBFOLIAGE 1.3493 1.0343 1.305 0.19206

FDSUBGROUND 3.0795 1.0347 2.976 0.00292 **

ORDERPiciformes -2.1789 1.1489 -1.897 0.05788 .

---

Significance codes: 0 '***' 0.001 '**' 0.01 '*' 0.05 '.' 0.1 ' ' 1

Null deviance: 394.24 on 619 degrees of freedom

Residual deviance: 333.14 on 614 degrees of freedom

Reference levels are YEAR = 2003, (feeding substrate) FDSUB = AERIAL, and ORDER = Passeriformes. Intercept, year, and feeding substrate = BARK and GROUND are highly significant predictors of presence of larvae on birds. ORDER = Piciformes is also significant, and negatively correlates with larvae present on birds.

**Tables E.**  Model selection on second-order Akaike Information Criterion (AICc) values for binomial models of bird life history traits and sampling conditions that lead to *I. pacificus* **larvae presence** on a bird, limited to the order Passeriformes.

| **Model** | **K** | **AICc** | **Δ AICc** | **AICcWt** | **Cum.Wt** | **LL** |
| --- | --- | --- | --- | --- | --- | --- |
| y ~ YEAR + NEST + FDSUB | 12 | 329.00 | 0.00 | 0.89 | 0.89 | -152.23 |
| y ~ YEAR + FDSUB | 5 | 335.42 | 6.42 | 0.04 | 0.92 | -162.66 |
| y ~ YEAR + MNFD + FDSUB | 7 | 335.60 | 6.59 | 0.03 | 0.95 | -160.70 |
| y ~ YEAR + FDSUB + RESSTAT | 6 | 336.66 | 7.66 | 0.02 | 0.97 | -162.26 |
| y ~ YEAR + RESSTAT + FDSUB | 6 | 336.66 | 7.66 | 0.02 | 0.99 | -162.26 |
| y ~ YEAR + MNHAB + FDSUB | 9 | 339.71 | 10.71 | 0.00 | 1.00 | -160.70 |

Evidence ratio between top model and next best-supported model: 24.78

For the best-supported model: glm(formula = y ~ YEAR + NEST + FDSUB, family = binomial) (where y = c[with larvae, without larvae]), model results are summarized below:

**Estimate Std. Error z value Pr(>|z|)**

(Intercept) 2987.3824 732.9353 4.076 4.58e-05 ***

YEAR -1.4926 0.3659 -4.080 4.51e-05 ***

cNESTCAVITY -1.1784 1.3407 -0.879 0.37944

cNESTCLIFF -14.1921 965.9214 -0.015 0.98828

cNESTGROUND -1.8223 1.4556 -1.252 0.21058

cNESTNON -1.7831 1.5753 -1.132 0.25765

cNESTOPP -16.7246 2399.5452 -0.007 0.99444

cNESTSHRUB -2.9911 1.5028 -1.990 0.04655 *

cNESTTREE -2.9082 1.4315 -2.032 0.04219 *

FDSUBBARK 2.5556 1.3145 1.944 0.05188 .

FDSUBFOLIAGE 1.9301 1.0517 1.835 0.06646 .

FDSUBGROUND 3.8586 1.1589 3.330 0.00087 ***

**---**

Significance codes: 0 '***' 0.001 '**' 0.01 '*' 0.05 '.' 0.1 ' ' 1

Null deviance: 383.39 on 588 degrees of freedom

Residual deviance: 304.46 on 577 degrees of freedom

Reference levels are YEAR = 2003, (nest placement for breeding birds) cNEST = BARK, (feeding substrate) FDSUB = AERIAL. Intercept, year, and feeding substrate = GROUND are highly significant predictors of presence of larvae on birds. Nest placement in trees and shrubs are significant and predict lower prevalences of larval infestation on birds.

**Tables F.**  Model selection for bird traits and sampling conditions that lead to *Ixodes pacificus* **nymph presence** on a bird, including the orders Passeriformes and Piciformes.

| **Model** | **K** | **AICc** | **Δ AICc** | **AICcWt** | **Cum.Wt** | **LL** |
| --- | --- | --- | --- | --- | --- | --- |
| y ~ YEAR + FAMILY | 23 | 321.57 | 0.00 | 0.99 | 0.99 | -136.86 |
| y ~ FAMILY | 22 | 331.54 | 9.97 | 0.01 | 1.00 | -142.92 |
| y ~ YEAR + NEST + MNFD | 11 | 346.99 | 25.42 | 0.00 | 1.00 | -162.28 |

Evidence ratio between top model and next best-supported model: 146.47

For the best-supported model: glm(formula = y ~ YEAR + FAMILY, family = binomial) (where y = c[with nymphs, without nymphs]), model results are summarized below:

**Estimate Std. Error z value Pr(>|z|)**

(Intercept) 2.404e+03 2.437e+03 0.986 0.32390

YEAR -1.210e+00 3.630e-01 -3.333 0.00086 ***

FAMILYCardinalidae 1.818e+01 2.326e+03 0.008 0.99376

FAMILYCerthiidae 2.091e+01 2.326e+03 0.009 0.99283

FAMILYCorvidae 2.036e+01 2.326e+03 0.009 0.99302

FAMILYEmberizidae 1.812e+01 2.326e+03 0.008 0.99378

FAMILYFringillidae 1.714e+01 2.326e+03 0.007 0.99412

FAMILYHirundinidae 5.568e-01 4.200e+03 0.000 0.99989

FAMILYIcteridae 1.829e+01 2.326e+03 0.008 0.99372

FAMILYMimidae 1.927e+01 2.326e+03 0.008 0.99339

FAMILYParidae 1.760e+01 2.326e+03 0.008 0.99396

FAMILYParulidae 1.718e-01 2.863e+03 0.000 0.99995

FAMILYPasseridae 5.612e-01 2.551e+03 0.000 0.99982

FAMILYPicidae 1.724e+01 2.326e+03 0.007 0.99409

FAMILYSittidae 9.886e-02 4.410e+03 0.000 0.99998

FAMILYSturnidae 1.086e+00 1.100e+04 0.000 0.99992

FAMILYSylviidae 3.564e-01 7.780e+03 0.000 0.99996

FAMILYThraupidae 9.886e-02 5.786e+03 0.000 0.99999

FAMILYTimaliidae 2.994e-01 3.037e+03 0.000 0.99992

FAMILYTroglodytidae 1.936e+01 2.326e+03 0.008 0.99336

FAMILYTurdidae 1.868e+01 2.326e+03 0.008 0.99359

FAMILYTyrannidae 1.611e+01 2.326e+03 0.007 0.99447

FAMILYVireonidae 1.724e+01 2.326e+03 0.007 0.99408

---

Significance codes: 0 '***' 0.001 '**' 0.01 '*' 0.05 '.' 0.1 ' ' 1

Null deviance: 366.75 on 619 degrees of freedom

Residual deviance: 273.71 on 597 degrees of freedom

Reference levels are YEAR = 2003, and FAMILY = Aegithalidae. Intercept and year are highly significant predictors of presence of nymphs on birds. While none of the levels of FAMILY is significant by themselves, the inclusion of the variable FAMILY is responsible for best model fits. On inspection, most instances of nymphs removed from birds come from FAMILY levels Certhiidae, Corvidae, and Troglodytidae, however, the taxonomic Families are not populated enough with data from this study to yield significance. Better significance might be obtained with sample sizes of >15 data instances per Family. Because Genus has more levels than Family, it does not appear that the model has been overfit to the category having the greatest number of levels. Although there is no statistical significance to the variable “FAMILY” in this model, the model selection results suggest that *I. pacificus* nymphs may be able to focus in on some taxonomic Families of birds more easily than others. This question remains open for future research.

**Tables G.**  Model selection for bird traits and sampling conditions that result in *Ixodes pacificus* **nymph presence** on a bird, limited to the order Passeriformes.

| **Model** | **K** | **AICc** | **Δ AICc** | **AICcWt** | **Cum.Wt** | **LL** |
| --- | --- | --- | --- | --- | --- | --- |
| y ~ YEAR + FAMILY + MNHAB | 26 | 303.02 | 0.00 | 0.36 | 0.36 | -124.26 |
| y ~ YEAR + FAMILY | 22 | 304.06 | 1.04 | 0.22 | 0.58 | -129.13 |
| y ~ YEAR + FAMILY + MNHAB + FDSUB | 28 | 304.35 | 1.33 | 0.19 | 0.76 | -122.72 |
| y ~ YEAR + FAMILY + FDSUB | 24 | 305.93 | 2.91 | 0.08 | 0.85 | -127.90 |
| y ~ YEAR + FAMILY + RESSTAT | 23 | 306.21 | 3.19 | 0.07 | 0.92 | -129.13 |
| y ~ YEAR + FAMILY + MNHAB + FDSUB + RESSTAT | 29 | 306.39 | 3.37 | 0.07 | 0.99 | -122.64 |
| y ~ YEAR + FDSUB + NEST | 12 | 310.57 | 7.56 | 0.01 | 1.00 | -143.02 |
| y ~ YEAR + LOG.AVEBWT + NEST | 10 | 312.21 | 9.19 | 0.00 | 1.00 | -145.92 |
| y ~ FAMILY | 21 | 314.64 | 11.62 | 0.00 | 1.00 | -135.51 |

Evidence ratio between top model and next best-supported model: 1.68

For the best-supported model: glm(formula = y ~ YEAR + FAMILY + MNHAB, family = binomial) (where y = c[with nymphs, without nymphs]), results are summarized below:

**Estimate Std. Error z value Pr(>|z|)**

(Intercept) 2514.0779 2453.4565 1.025 0.30550

YEAR -1.2649 0.3911 -3.234 0.00122 **

FAMILYCardinalidae 16.7558 2325.0514 0.007 0.99425

FAMILYCerthiidae 19.1139 2325.0514 0.008 0.99344

FAMILYCorvidae 19.2696 2325.0512 0.008 0.99339

FAMILYEmberizidae 16.8835 2325.0511 0.007 0.99421

FAMILYFringillidae 15.3368 2325.0514 0.007 0.99474

FAMILYHirundinidae 0.1150 4195.1233 0.000 0.99998

FAMILYIcteridae 17.5096 2325.0514 0.008 0.99399

FAMILYMimidae 19.3179 2325.0513 0.008 0.99337

FAMILYParidae 17.1393 2325.0512 0.007 0.99412

FAMILYParulidae -1.6325 2861.7118 -0.001 0.99954

FAMILYPasseridae 0.1196 2550.1640 0.000 0.99996

FAMILYSittidae -1.7076 4407.4493 0.000 0.99969

FAMILYSturnidae -0.1209 11002.4842 0.000 0.99999

FAMILYSylviidae -1.4414 7772.0407 0.000 0.99985

FAMILYThraupidae -1.7076 5783.1960 0.000 0.99976

FAMILYTimaliidae 0.3088 3034.6876 0.000 0.99992

FAMILYTroglodytidae 18.9304 2325.0512 0.008 0.99350

FAMILYTurdidae 18.1242 2325.0513 0.008 0.99378

FAMILYTyrannidae 14.8885 2325.0513 0.006 0.99489

FAMILYVireonidae 16.1115 2325.0512 0.007 0.99447

MNHABGRASS 1.2591 1.3083 0.962 0.33588

MNHABN/A -14.9734 10754.0130 -0.001 0.99889

MNHABOAKW 0.4617 1.0309 0.448 0.65427

MNHABXW 1.8093 0.8587 2.107 0.03512 *

---

Significance codes: 0 '***' 0.001 '**' 0.01 '*' 0.05 '.' 0.1 ' ' 1

Null deviance: 351.69 on 588 degrees of freedom

Residual deviance: 248.52 on 563 degrees of freedom

Reference levels are YEAR = 2003, FAMILY = Aegithalidae, and the main habitat MNHAB = CHAP (chaparral). When models are limited to the order Passeriformes here, similar results to those from the previous analysis with two bird Orders are obtained, in that YEAR and FAMILY are selected in the top model, with YEAR as a significant factor, and no level of FAMILY by itself showing up as significant. The variable MNHAB becomes important in the Passeriformes only model group, with MNHAB = XW (main habitat = dense oak woodland) exhibiting significance, as predicted. All levels of main habitat show positive correlations with nymphal presence on birds compared to MNHAB = chaparral except for MNHAB = N/A, which refers only to the Red-eyed Vireo, an unusual visitor with no known habitat preferences in this region.
