# Supplementary material for: Borrelia burgdorferi Sensu Lato Spirochetes in Wild Birds in Northwestern California: Associations with Ecological Factors, Bird Behavior and Tick Infestation
Source: PLoS One. 2015 Feb 25;10(2):e0118146. doi: 10.1371/journal.pone.0118146 (PMC4340631; doi:10.1371/journal.pone.0118146)
Supplement: S3 File — Count models, model selection AIC values, and parameter estimates for zero-inflated negative binomial models of larval and nymphal infestations of birds Tables H. Model selection AIC values for bird life history traits that predict number of Ixodes pacificus larvae removed from a bird, including the orders Passeriformes and Piciformes Tables I. Model selection AIC values for bird life history traits that lead to number of Ixodes pacificus nymphs removed from a bird, including the orders Passeriformes and Piciformes (DOCX) [file pone.0118146.s003.docx]

**Supporting Information**

**File S3.** Count models, model selection AIC values, and parameter estimates for zero-inflated negative binomial models of larval and nymphal infestations of birds

For zero-inflated negative binomial models, overdispersion can be accommodated. Unlike Poisson models, support is finite. Models that were singular when estimated and many non-significant models are not listed.

**Tables H.** Model selection AIC values for bird life history traits that predict **number of *Ixodes pacificus* larvae removed from a bird**, including the orders Passeriformes and Piciformes. Models consist of a negative binomial count model with a log link, and a zero-inflation binomial model with a logit link, in the form y ~ (count model | zero-inflated model). Here, y = number of larvae removed from an individual bird.

| **Model specification** | **K** | **AIC value** | **Δ AIC** | **AIC_Wt** | **Cum.Wt** |
| --- | --- | --- | --- | --- | --- |
| y ~ MNHAB + FDSUB \| YEAR + FDSUB | 13 | 514.81 | 0 | 0.64 | 0.64 |
| y ~ MNHAB + FDSUB \| YEAR + RESSTAT + FDSUB | 14 | 516.33 | 1.52 | 0.30 | 0.94 |
| y ~ MNHAB + FDSUB \| YEAR | 10 | 519.76 | 4.95 | 0.05 | 1.00 |
| y ~ MNHAB \| FDSUB | 9 | 527.79 | 12.98 | 0.00 | 1.00 |

For the top supported model (y ~ MNHAB + FDSUB | YEAR + FDSUB), **count model** variables, levels, and results are summarized below:

**Estimate Std. Error z value Pr(>|z|)**

(Intercept) -3.849967 2.713162 -1.419 0.15590

MNHABGRASS 4.475914 0.689302 6.493 8.39e-11 ***

MNHABOAKW 1.183432 0.527190 2.245 0.02478 *

MNHABXW 1.395131 0.506296 2.756 0.00586 **

FDSUBBARK 1.611221 2.860301 0.563 0.57323

FDSUBFOLIAGE 2.339049 2.720155 0.860 0.38985

FDSUBGROUND 2.475747 2.702043 0.916 0.35954

Log(theta) 0.005238 0.363146 0.014 0.98849

**Zero-inflation model** (binomial with logit link) variables, levels and results are summarized below:

**Estimate Std. Error z value Pr(>|z|)**

(Intercept) -3504.9835 3.5846 -977.780 <2e-16 ***

YEAR 1.7502 NA NA NA

FDSUBBARK -0.8613 3.9145 -0.220 0.826

FDSUBFOLIAGE 0.5193 3.5910 0.145 0.885

FDSUBGROUND -1.5068 3.5902 -0.420 0.675

Theta = 1.0053

**---**

Significance codes: 0 '***' 0.001 '**' 0.01 '*' 0.05 '.' 0.1 ' ' 1

Reference levels are YEAR = 2003, (main habitat) MNHAB = CHAP (chaparral), and (feeding substrate) FDSUB = AERIAL. Main habitat = grass, oak woodland, and dense oak woodland are all significant in predicting higher numbers of larvae on birds compared to the reference level, chaparral. Intercept is highly significant in predicting presence of larvae on birds.

**Tables I.** Model selection AIC values for bird life history traits that lead to **number of *Ixodes pacificus* nymphs removed from a bird**, including the orders Passeriformes and Piciformes. Models consist of a negative binomial count model with a log link, and a zero-inflation binomial model with a logit link, in the form y ~ (count model | zero-inflated model). Here, y = number of nymphs removed from an individual bird.

| **Model specification** | **K** | **AIC value** | **Δ AIC** | **AIC_Wt** | **Cum.Wt** |
| --- | --- | --- | --- | --- | --- |
| y ~ MNHAB \| LOG.AVEBWT + YEAR | 8 | 464.51 | 0.00 | 0.65 | 0.65 |
| y ~ MNHAB + FDSUB \| YEAR + FDSUB | 13 | 467.46 | 2.95 | 0.15 | 0.80 |
| y ~ MNHAB + FDSUB \| YEAR + RESSTAT + FDSUB | 14 | 468.82 | 4.30 | 0.08 | 0.88 |
| y ~ MNHAB + FDSUB \| YEAR | 10 | 468.90 | 4.39 | 0.07 | 0.95 |
| y ~ YEAR + MNHAB + LOG.AVEBWT \| YEAR + ORDER | 10 | 471.64 | 7.12 | 0.02 | 0.97 |
| y ~ YEAR + MNHAB \| YEAR + ORDER | 9 | 472.13 | 7.61 | 0.01 | 0.98 |
| y ~ MNHAB \| FDSUB | 9 | 473.11 | 8.60 | 0.01 | 0.99 |
| y ~ MNHAB + FDSUB \| 1 | 9 | 474.99 | 10.48 | 0.00 | 0.99 |
| y ~ MNHAB + FDSUB \| RESSTAT + FDSUB | 13 | 476.40 | 11.89 | 0.00 | 1.00 |
| y ~ FDSUB \| LOG.AVEBWT + YEAR | 8 | 477.21 | 12.69 | 0.00 | 1.00 |

For the top supported model (y ~ MNHAB | LOG.AVEBWT + YEAR), **count model** variables, levels, and model results are summarized below:

**Estimate Std. Error z value Pr(>|z|)**

(Intercept) -2.5687 0.6963 -3.689 0.000225 ***

MNHABGRASS 3.4812 0.9826 3.543 0.000396 ***

MNHABOAKW 1.3148 0.6724 1.956 0.050514 .

MNHABXW 1.8163 0.6871 2.644 0.008204 **

Log(theta) -0.8458 0.5171 -1.636 0.101891

**Zero-inflation model** (binomial with logit link) variables, levels and P-values are:

**Estimate Std. Error z value Pr(>|z|)**

(Intercept) -2804.3936 0.9822 -2855.354 <2e-16 ***

LOG.AVEBWT -2.3778 1.1848 -2.007 0.0448 *

YEAR 1.4014 NA NA NA

Theta = 0.4292

**---**

Significance codes: 0 '***' 0.001 '**' 0.01 '*' 0.05 '.' 0.1 ' ' 1

Reference levels are YEAR = 2003, (main habitat) MNHAB = CHAP (chaparral), and (feeding substrate) FDSUB = AERIAL. Main habitat = grass, oak woodland, and dense oak woodland are all significant in predicting higher numbers of nymphs on birds compared to the reference level, chaparral. Intercept and log (average bodyweight) are significant in predicting presence of nymphs on birds.
